# Trial of Early Antiviral Therapies during Non-hospitalized Outpatient Window (TREAT NOW) for COVID-19: a summary of the protocol and analysis plan for a decentralized randomized controlled trial

**DOI:** 10.1186/s13063-022-06213-z

**Published:** 2022-04-08

**Authors:** Alexander M. Kaizer, Jessica Wild, Christopher J. Lindsell, Todd W. Rice, Wesley H. Self, Samuel Brown, B. Taylor Thompson, Kimberly W. Hart, Clay Smith, Michael S. Pulia, Nathan I. Shapiro, Adit A. Ginde

**Affiliations:** 1 Department of Biostatistics and Informatics, Colorado School of Public Health, University of Colorado, Aurora, USA; 2 Department of Biostatistics, Vanderbilt University, Nashville, USA; 3 Division of Allergy, Pulmonary, and Critical Care Medicine, Vanderbilt University Medical Center, Nashville, USA; 4 Department of Emergency Medicine and Vanderbilt Institute for Clinical and Translational Research, Vanderbilt University Medical Center, Nashville, USA; 5 Division of Pulmonary and Critical Care Medicine, Department of Medicine, Intermountain Medical Center, Salt Lake City, USA; 6 Division of Pulmonary and Critical Care Medicine, Department of Internal Medicine, University of Utah School of Medicine, Salt Lake City, USA; 7 Division of Pulmonary and Critical Care Medicine, Department of Medicine, Massachusetts General Hospital and Harvard Medical School, Boston, USA; 8 Department of Emergency Medicine, Vanderbilt University Medical Center, Nashville, USA; 9 Departments of Emergency Medicine and Industrial and Systems Engineering, University of Wisconsin-Madison, Madison, USA; 10 Department of Emergency Medicine, Beth Israel Deaconess Medical Center and Harvard Medical School, Boston, USA; 11 Department of Emergency Medicine, University of Colorado School of Medicine, Aurora, USA

**Keywords:** COVID-19, Outpatient, Protocol, Randomized, Remote, Antiviral

## Abstract

**Background:**

Coronavirus disease 2019 (COVID-19) has a heterogeneous outcome in individuals from remaining asymptomatic to death. In a majority of cases, mild symptoms are present that do not require hospitalization and can be successfully treated in the outpatient setting, though symptoms may persist for a long duration. We hypothesize that drugs suitable for decentralized study in outpatients will have efficacy among infected outpatients

**Methods:**

The TREAT NOW platform is designed to accommodate testing multiple agents with the ability to incorporate new agents in the future. TREAT NOW is an adaptive, blinded, multi-center, placebo-controlled superiority randomized clinical trial which started with two active therapies (hydroxychloroquine and lopinavir/ritonavir) and placebo, with the hydroxychloroquine arm dropped shortly after enrollment began due to external evidence. Each arm has a target enrollment of 300 participants who will be randomly assigned in an equal allocation to receive either an active therapy or placebo twice daily for 14 days with daily electronic surveys collected over days 1 through 16 and on day 29 to evaluate symptoms and a modified COVID-19 ordinal outcome scale. Participants are enrolled remotely by telephone and consented with a digital interface, study drug is overnight mailed to study participants, and data collection occurs electronically without in-person interactions.

**Discussion:**

If effective treatments for COVID-19 can be identified for individuals in the outpatient setting before they advance to severe disease, it will prevent progression to more severe disease, reduce the need for hospitalization, and shorten the duration of symptoms. The novel decentralized, “no touch” approach used by the TREAT NOW platform has distinction advantages over traditional in-person trials to reach broader populations and perform study procedures in a pragmatic yet rigorous manner.

**Trial registration:**

ClinicalTrials.gov NCT04372628. Registered on April 30, 2020. First posted on May 4, 2020.

**Supplementary Information:**

The online version contains supplementary material available at 10.1186/s13063-022-06213-z.

## Administrative information

Note: the numbers in curly brackets in this protocol refer to SPIRIT checklist item numbers. The order of the items has been modified to group similar items (see http://www.equator-network.org/reporting-guidelines/spirit-2013-statement-defining-standard-protocol-items-for-clinical-trials/).

**Table:** 

Title {1}	Trial of Early Antiviral Therapies during Non-hospitalized Outpatient Window (TREAT NOW) for COVID-19: A Summary of the Protocol and Analysis Plan for a Decentralized Randomized Controlled Trial
Trial registration {2a and 2b}.	ClinicalTrials.gov NCT04372628. Registered on April 30, 2020. First posted on May 4, 2020.
Protocol version {3}	Version 1.6 (January 26, 2021)
Funding {4}	The U.S. Department of Defense (ID07200010-301-2) and AbbVie
Author details {5a}	AMK, JW: University of Colorado, Colorado School of Public Health, Department of Biostatistics and Informatics AAG: University of Colorado School of Medicine, Department of Emergency Medicine MSP: University of Wisconsin-Madison, Departments of Emergency Medicine and Industrial and Systems Engineering TWR: Vanderbilt University Medical Center, Division of Allergy, Pulmonary, and Critical Care Medicine WHS: Vanderbilt University Medical Center, Department of Emergency Medicine and Vanderbilt Institute for Clinical and Translational Research CS: Vanderbilt University Medical Center, Department of Emergency Medicine NIS: Beth Israel Deaconess Medical Center and Harvard Medical School, Department of Emergency Medicine. SB: Division of Pulmonary and Critical Care Medicine, Department of Medicine, Intermountain Medical Center, and Division of Pulmonary and Critical Care Medicine, Department of Internal Medicine, University of Utah School of Medicine CJL, KWH: Vanderbilt University, Department of Biostatistics BTT: Division of Pulmonary and Critical Care Medicine, Department of Medicine, Massachusetts General Hospital and Harvard Medical School
Name and contact information for the trial sponsor {5b}	Vanderbilt University Medical Center Vanderbilt Coordinating Center 2525 West End Ave, Suite 1440 | Nashville, TN 37203 Phone: 615-343-6485
Role of sponsor {5c}	TREAT NOW is an academic, investigator–initiated and -led study by the (co-)authors. Funding was obtained from the Department of Defense and AbbVie pharmaceuticals. None of the funders was involved in any of the following: study design; collection, management, analysis, and interpretation of data; writing of the report; and the decision to submit the report for publication, including whether they will have ultimate authority over any of these activities

## Introduction

### Background and rationale {6a}

Coronavirus disease 2019 (COVID-19) is an acute respiratory infectious illness caused by severe acute respiratory syndrome coronavirus 2 (SARS-CoV-2) [1, 2]. Most adults with COVID-19 appear to experience fever, cough, and fatigue and then recover within 1–3 weeks, but this can be affected by underlying risk factors [3, 4]. However, up to 15% of adults with COVID-19 develop severe illness, typically manifesting as pneumonia and hypoxemic respiratory failure, with continued progression to acute respiratory distress syndrome (ARDS) and death in some cases [1, 2, 5, 6]. There is an urgent need to identify multiple outpatient therapies with a demonstrated ability to prevent the progression of COVID-19 to severe illness and to improve recovery.

The purpose of this prospective randomized trial is to evaluate the potential effectiveness of candidate therapies for use in the outpatient setting that can reduce the likelihood of experiencing progression of COVID-19 to severe illness. The TREAT NOW platform focuses on repurposed medications, with a focus on antivirals, which have FDA approval for other indications, and have shown promise in COVID-19. Based on the mechanism of action and early clinical experiences, several agents currently available in the U.S. are proposed as potential therapies to prevent disease progression [7–9]. Among these potential therapies, lopinavir/ritonavir has generated substantial interest due to antiviral and immunomodulatory activity and established safety profiles with FDA approval for use in other conditions. Hydroxychloroquine was initially included as another active therapy arm, but was dropped shortly after trial enrollment began due to external evidence on its lack of efficacy [10–14]. Therefore, in the first stage of TREAT NOW, we will evaluate the effectiveness and safety of lopinavir/ritonavir for the early treatment of adults with COVID-19 in the outpatient setting, prior to hospitalization. However, given the platform nature of the study, it is possible to introduce new arms as the trial is ongoing.

### Objectives {7}

The overall objective of TREAT NOW is to determine the effectiveness and safety of early treatment with lopinavir/ritonavir versus placebo control in outpatient adults with COVID-19. A placebo control is used rather than active control given the lack of prior evidence of effective therapies in the outpatient setting to prevent the progression of COVID-19 to severe disease. The primary hypothesis of TREAT NOW is that early initiation of lopinavir/ritonavir will reduce disease progression and improve clinical outcomes among outpatient adults with COVID-19.

### Trial design {8}

TREAT NOW is a decentralized platform trial using a common infrastructure, data collection methodology, and analysis approach for evaluating the effectiveness of repurposed agents for preventing the progression of mild to moderate outpatient Covid-19, and for enhancing recovery. TREAT NOW is an adaptive, blinded, multi-center, placebo-controlled, randomized clinical trial being conducted within the overall TREAT NOW platform. The TREAT NOW study is a Phase 2b superiority trial evaluating lopinavir/ritonavir vs placebo in the early outpatient treatment of adults with COVID-19. There is equal allocation between study arms with stratification by center and age (≥ 65 years or < 65 years), with the randomization sequence stored securely with the allocation known only to the investigational pharmacy and unblinded statistical team.

## Methods: participants, interventions, and outcomes

### Study setting {9}

TREAT NOW is an outpatient multi-center study with enrollment restricted to the USA. The participating centers at the time of this writing are:

- Vanderbilt University Medical Center (Nashville, TN)
- University of Colorado School of Medicine (Aurora, CO)
- Beth Israel Deaconess Medical Center (Boston, MA)
- University of Mississippi Medical Center (Jackson, MS)
- University of Wisconsin-Madison (Madison, WI)

Any updates to the list of study sites will be included on ClinicalTrials.gov. It is important to note that, given the outpatient nature of this study and that treatment does not take place at a given site, an individual site enrolls patients out of state and therefore enrollment occurs nationally based upon meeting the eligibility criteria outlined in the following section.

### Eligibility criteria {10}

Eligible participants must be 18 years of age or older with a laboratory-confirmed SARS-CoV-2 infection by RT-PCR or other molecular tests, or by antigen test with emergency use authorization or full approval collected within the past 6 days. Additionally, participants must have had at least one of the following symptoms of acute respiratory infection for six or fewer days:

- Cough
- Fever
- Shortness of breath
- Chest pain
- Abdominal pain
- Nausea/vomiting
- Diarrhea
- Body aches
- Weakness/fatigue

The full exclusion criteria are listed in the protocol (see Additional file 1); however, we briefly summarize some aspects here. First, only one individual can be enrolled from the same household to prevent potential unblinding of treatment assignment since the placebo is not matched to the lopinavir/ritonavir pill. Second, the inability to participate in English or Spanish for the daily symptom/safety survey will result in exclusion since survey materials are only available in these two languages. Third, those previously enrolled in the trial will not be allowed to re-enroll for subsequent or recurring infection. Finally, a number of medications that may interact with study treatment are identified and listed in the full protocol; a conservative approach whereby patients with potentially interacting medications are excluded given remote enrollment and the intrinsic lack of baseline electrocardiogram and laboratory tests.

### Who will take informed consent? {26a}

Potential trial participants will be contacted by study staff from their respective centers if they appear to meet the initial criteria of a positive molecular test and are not currently hospitalized for further screening. Individuals not receiving direct care at one of the participating centers may become aware of the trial from ClinicalTrials.gov, targeted advertising on internet platforms for select geographic areas (e.g., Google and Facebook), or other means are able to contact study staff at the coordinating center at Vanderbilt to be screened for enrollment. Individuals who reach out in response to advertising are assigned to one of the study centers for contact.

Written informed consent will be obtained from the participant. Participants who lack decision-making capacity will not be enrolled due to logistical issues with remote study medication administration, safety monitoring, and accurate data collection. In general, we will use “no-touch” consent procedures for this trial, employing an electronic remote consent process to obtain written informed consent. This electronic approach is as follows:

1. A link for the electronic consent is sent to the subject.
2. Research staff contact the patient by telephone or videophone (method dictated by institutional policy) to have an informed consent conversation. This step confirms subject identity.
3. If they consent, the patient signs the consent form. This can be:
  - An actual signature (often tracing their finger on the screen) OR
  - A username and password specific to the individual signing

This approach complies with relevant regulations and sub-regulator guidance at 45 CFR 46.117, 45 CFR 164.512, 21 CFR 11 Subpart C (11.100–11.300).

We will provide the information for the informed consent discussion in a formal document that has been approved by the IRB and in a language comprehensible to the potential participant, using an interpreter if necessary. Currently, English and Spanish documents are approved for use in this trial. The information presented in the consent form and by the research staff will detail the nature of the trial and what is expected of participants, including any potential risks or benefits of taking part. We will clearly state that the participant is free to withdraw from the trial at any time for any reason without prejudice to future care, and with no obligation to give the reason for withdrawal. Where a patient does not speak English, a translated Spanish consent and qualified interpreter will be employed, using similar “no-touch” principles. The use of a telephone or video interpreter and the interpreter’s identity will be documented on the electronic consent. After allowing the potential participant time to read the informed consent document, the research staff will answer any additional questions.

### Additional consent provisions for collection and use of participant data and biological specimens {26b}

Not applicable, no biological specimens are collected.

## Interventions

### Explanation for the choice of comparators {6b}

At the time of protocol development, there were no approved therapies for treating COVID-19 in an outpatient setting. Thus, the comparator to the active lopinavir/ritonavir arm under investigation is a placebo control instead of an active control. If a therapeutic agent becomes approved in the future, a future arm may include a yet-to-be-defined active comparator instead of the currently selected placebo comparator.

### Intervention description {11a}

The first two treatments selected for comparison to placebo were hydroxychloroquine and lopinavir/ritonavir. As noted earlier, the hydroxychloroquine arm was terminated due to external evidence for lack of efficacy, so we focus on describing lopinavir/ritonavir below.

Participants assigned to the lopinavir/ritonavir arm will receive lopinavir/ritonavir 800 mg/200 mg orally twice daily for two doses (day 1), then 400 mg/100 mg twice daily for the subsequent 26 doses (days 2–14). This dosing regimen is a total of 28 doses over 14 days with a 1600 mg/400 mg load on the first day divided into two doses followed by 800 mg/200 mg daily divided into two doses over the following 13 days. Medication dose packs will contain all 28 doses labeled by study day.

Participants randomized to the control group will receive a placebo orally twice daily for 14 days. Medication dose packs will contain all 28 doses labeled by study day.

TREAT NOW uses a central pharmacy located in Colorado to ship the allocated treatment to randomized study participants through overnight mail to standardize treatment delivery and minimize contact. Depending on the time of day for randomization and other potential shipping delays, it is possible medication will not be received the day after randomization. To account for this, “study day 1” is identified as the day when there is a receipt of study medication from the central pharmacy.

### Criteria for discontinuing or modifying allocated interventions {11b}

We will stop administration of the blinded study drug temporarily or permanently for (a) adverse events without evidence of an alternative cause to the patient’s symptoms, (b) results of on-study monitoring based on any in-person interactions with a healthcare provider and reported during the daily self-report surveys, or (c) clinical deterioration if requested by treating clinician. If an electrocardiogram (EKG) is obtained as part of routine clinical care after enrollment and the QT interval correct for heart rate (QTc) is greater than or equal to 500 ms, we will discontinue the study drug, unless a repeat EKG after at least 24 h demonstrates the QTc is less than 500 ms, at which time we will direct the patient to resume the study drug. Other criteria are further described in the full protocol (see Additional file 1).

### Strategies to improve adherence to interventions {11c}

Adherence to assigned intervention is encouraged through self-reported daily assessments to evaluate safety and efficacy over the course of the 14-day treatment period. Study coordinators will directly reach out if a participant reports pausing or permanently stopping study medication prior to completion of all 28 doses.

### Relevant concomitant care permitted or prohibited during the trial {11d}

Enrolled participants will not receive open-label lopinavir/ritonavir during the 14-day intervention period, unless the patient is hospitalized, and the treating clinician wishes to unblind the trial and use these medications open label. The treating clinicians will make all other treatment decisions without influence from the protocol. Administration of other antiviral, immunomodulatory, or other COVID-19 directed novel medications (“rescue therapy”) will be allowed. We will record the co-administered COVID-19 directed medications in the case report form. The patient will be allowed to take antipyretics at home as needed.

### Provisions for post-trial care {30}

Since participants continue to receive standard of care from their own physician or medical team, there is no formal plan for post-trial care after completion of all study surveys and measures.

### Outcomes {12}

The primary outcome of TREAT NOW is a modified COVID-19 ordinal outcomes scale measured longitudinally through day 15 of the study:

1. Death
2. Hospitalized on mechanical ventilation or ECMO
3. Hospitalized on supplemental oxygen
4. Hospitalized not on supplemental oxygen
5. Not hospitalized with symptoms and limitation in activity
6. Not hospitalized with symptoms but with no limitation in activity
7. Not hospitalized without symptoms nor limitation in activity

The scale was chosen as it was initially proposed by the World Health Organization for COVID-19 studies early in the pandemic [15]. However, given that the TREAT NOW platform focuses on an outpatient population, we expect to have less severe outcomes overall and modified the proposed ordinal scale to capture greater nuance in the status of those not hospitalized. Limitations are defined based on participant self-report with identification if reported symptoms limit normal daily activities and are therefore relative for each participant.

Secondary outcomes are briefly defined below, with further definitions for how each will be operationalized in the statistical analysis plan (see Additional file 2):

- Modified COVID Ordinal Outcome Scale to study day 8
- Modified COVID Ordinal Outcome Scale to study say 29
- Proportion of patients hospitalized to study day 29
- Time to hospitalization to study day 29
- Time to symptom resolution to study day 29
- All-cause, all-location mortality to study day 29
- Oxygen-free days through study day 29
- Fever-free days to study day 29
- Ventilator-free days through study day 29
- Vasopressor-free days through study day 29
- Intensive care unit (ICU)-free days through study day 29
- Hospital-free days through study day 29

Safety outcomes to be included in the safety analysis for this trial include all potentially associated adverse events (PAAEs) as well as other events of interest including, but not necessarily limited to:

- Seizure
- Atrial or ventricular arrhythmia
- Cardiac arrest
- Receipt of renal replacement therapy
- Severe dermatologic reaction
*Elevation in aspartate aminotransferase or alanine aminotransferase to twice the local upper limit of normal AND at least doubling over known baseline
*Acute pancreatitis
*Acute kidney injury by KDIGO criteria
*Symptomatic hypoglycemia
*Anemia or thrombocytopenia

* For participants whose symptoms are significant enough to trigger a clinical work-up and thus have clinically available testing

## Participant timeline {13}

The participant timeline is shown in Table 1.

**Table 1:** Participant timeline

Description:	Date of randomization	Days 1–14	Days 15–16	Day 29
Confirmation of SAR-CoV-2 positive test result	X			
Home medications	X			
Verification of inclusion/exclusion criteria	X			
Remote consent	X			
Randomization	X			
Investigational product shipped to subject	X			
Self-administered dosing		X		
Self-Reported Symptom Survey		X	X	X
Assessment of adverse events		X	X	X
Modified COVID Ordinal Outcome Scale		X	X	X
Ventilator status		X	X	X
ICU status		X	X	X
Oxygen status		X	X	X
Hospital length of stay		X	X	X
Vital status				X
End of study				X

### Sample size {14}

When considering the pairwise comparison of an active therapy to a control arm, a total sample size of 540 patients equally allocated to both study arms is needed to have 90% power to detect an odds ratio of 1.75 or greater assuming a traditional proportional odds model and a type I error of 0.05, as proposed by Whitehead [16]. To account for an approximate 10% loss to follow-up rate, we plan to enroll up to 600 patients in TREAT NOW. Therefore, a sample size of 300 is targeted for each arm within the TREAT NOW platform. This sample size and power is based on conservative assumptions and a frequentist analysis. We discuss the proposed Bayesian analysis approach for the longitudinal outcome in a later section, noting that sufficient preliminary data were unavailable for estimating power using this approach at the time this study was designed and the Bayesian methodology was still being refined.

An example of the difference between study arms resulting in an odds ratio of 1.75 is shown in the following table. This is based on recommendations from the World Health Organization for COVID-19 Master Protocols that were in place at the time this study was designed (Table 2).

**Table 2:** Assumed response rates ordinal category for power calculation

Category	Placebo arm proportion	Treatment arm proportion
1-Death	1%	0.6%
2-Hospitalized on mechanical ventilation or ECMO	3%	1.8%
3-Hospitalized on supplemental oxygen	4%	2.4%
4-Hospitalized not on supplemental oxygen	3%	1.9%
5-Not hospitalized with symptoms and limitation in activity	10%	6.6%
6-Not hospitalized with symptoms but with no limitation in activity	24%	18.7%
7-Not hospitalized without symptoms nor limitation in activity	55%	68.1%

### Recruitment {15}

Patients will be recruited either through active outreach of individuals who test positive for the SARS-CoV-2 virus at one of our study centers or through a national advertising campaign. We established a study website (TREAT-NOW.org) and will use a national recruitment campaign on web-based platforms (e.g., Google and Facebook) to recruit eligible study participants who are not affiliated with one of the participating centers. This “national campaign” will broaden the recruitment population and allow study subjects to access research opportunities regardless of geographic constraints.

## Assignment of interventions: allocation

### Sequence generation {16a}

To maintain trial integrity and reproducibility, a randomization procedure through a central computerized system has been established. There is equal allocation across all active study arms in TREAT NOW, with stratification by site and age (≥ 65 years versus < 65 years).

### Concealment mechanism {16b}

A participant’s allocation is revealed electronically to the study staff after consent has been obtained and eligibility has been confirmed.

### Implementation {16c}

The allocation and computerized system is provided by Vanderbilt University. Participants are enrolled by study staff after written consent is obtained.

## Assignment of interventions: blinding

### Who will be blinded {17a}

The participant, treating clinicians, study personnel, and outcome assessors will all remain blinded to group assignment until after the database is locked and blinded analysis is completed. Only the central study pharmacy, who is distributing study medication, and one member of the biostatistical team who is preparing closed DSMB interim reports will be unblinded. Specifically, study medication will be dispensed with packaging and labeling that would blind treatment assignment. Unblinding will occur only if required for subject safety or treatment at the request of the treating clinician.

### Procedure for unblinding if needed {17b}

Unblinding will occur only if required for subject safety or treatment at the request of the treating clinician.

## Data collection and management

### Plans for assessment and collection of outcomes {18a}

Collection of information on outcomes, demographics, and other relevant summaries will be primarily collected through electronic means. Electronic data collection helps conduct the trial in a pragmatic manner and will increase the ability to complete the trial in the face of strained clinical and research resources during the COVID-19 pandemic and when in-person visits may be restricted by local regulations. If the patient is hospitalized, we will attempt to obtain permission for accessing these records.

We will collect data directly from patients primarily through text messaging or email with a survey link or phone call as backup. This provides the capacity for collecting patient-reported outcomes in a secure and robust manner. Our outcomes are designed such that a text messaging option is not overly burdensome and can even be completed in hospitalized patients. The process for using text messaging is HIPAA compliant. Finally, we can use standard telephone calls as a backup method to administer the questions. This minimizes loss to follow-up and maximizes the efficiency of the study. We will emphasize data collection by electronic methods, supplemented by telephone or videophone follow-up and from the electronic health record, with criteria for alternative methods further detailed in the protocol (see Additional file 1).

Baseline variables to be collected include presence or absence of each inclusion and exclusion criteria, date and time of enrollment, presence and duration of symptoms, date and result of SARS-CoV-2 molecular testing, demographics, comorbidities, current medication use, and self-reported severity of presenting signs and symptoms.

Using the REDCap system to deliver a symptom diary, daily assessments will take place from days 1 through 16 after randomization, as well as on day 29. These surveys will collect self-reported use of the study drug and reasons for missed doses; daily signs and symptoms with severity; receipt of antivirals, immunomodulators, or azithromycin; safety outcomes; and if hospitalized or using supplemental oxygen.

### Plans to promote participant retention and complete follow-up {18b}

If a participant does not respond to a daily assessment or indicates they are not continuing their study medication to completion, study coordinators will attempt to reach out to verify participant status for hospitalization, encourage continued participation, and identify where difficulties may be occurring in the process of collecting study-related measures.

### Data management {19}

The electronic data collection instruments will record responses directly into the REDCap database. This will help avoid issues with data quality relating to manually entering paper forms by study staff. All data will be securely stored in the REDCap database at Vanderbilt, which facilitates an auditable trail for any data modifications or access.

### Confidentiality {27}

Federal regulations at 45 CFR 46 111 (a) [7] require that when appropriate, there are adequate provisions to protect the privacy of participants and to maintain the confidentiality of data. At no time during the course of this study, its analysis, or its publication will we reveal patient identities in any manner. We will collect the minimum necessary data containing patient or provider identities. All patients will be assigned a unique study ID number for tracking. We will maintain all data in the secure online database until the time of study publication. At the time of publication, we will generate a de-identified version of the database. Further, we will use tools within the secure online database so that only the coordinating center and investigators from the enrolling site will have access to data from participants enrolled at that site.

### Plans for collection, laboratory evaluation, and storage of biological specimens for genetic or molecular analysis in this trial/future use {33}

No biological specimens are collected and no laboratory testing is done as part of this trial or for future use.

## Statistical methods

### Statistical methods for primary and secondary outcomes {20a}

The analyses for TREAT NOW will use the Bayesian paradigm to evaluate all outcomes for statistical inference. These approaches are summarized in the following section, but greater details can be found in the accompanying statistical analysis plan (see Additional Document Y).

We will compare the primary outcome (Modified COVID Ordinal Outcomes Scale score through Day 15) between patients in the intervention and control groups using a Bayesian longitudinal proportional odds model, adjusted for the following variables: age, gender, and duration of acute respiratory infection symptoms prior to randomization. To account for the longitudinally collected data within each participant, a random intercept will be included for each participant. The proportional odds assumption will be primarily examined using graphical methods—e. g., the logit of the empirical cumulative distribution function of the ordinal scale should be parallel among categories of covariates. If proportionality is clearly violated, we will consider partial proportional odds or non-proportional odds models.

The prior for the intercept for the proportional odds regression model will assume a Dirichlet distribution. The treatment effect, as defined as a log odds ratio in the model, will have a normal prior defined so that *P*(OR > 1) = *P*(OR < 1) = 0.5 (i.e., equally as harmful as beneficial) and $P\left(\mathrm{OR}>2\right)=P\left(\mathrm{OR}<\frac{1}{2}\right)=0.025$ (i.e., large effects in either direction unlikely), with the variance computed to satisfy these criteria. For all other covariates, the prior distributions will be normal with mean 0 and a larger variance to reflect the uncertainty of their potential effect. The convergence of the Markov chain Monte Carlo simulations will be checked via diagnostics, with alternative priors, models, and chain lengths/burn-ins explored if necessary to achieve convergence.

For secondary outcomes, we briefly denote the modeling approaches that will be considered. For time-to-event outcomes, we will utilize survival models (e.g., Cox proportional hazards models), and dichotomized outcomes will be evaluated using logistic regression. The continuous outcomes such as hospital-free days are penalized for death and so an ordinal model will be fit in preference to using linear regression models. Secondary endpoint models will be fit using normal priors with mean 0 and larger variance to reflect the uncertainty of their potential effect on all regression coefficients, with convergence evaluated similarly to the primary endpoint.

### Interim analyses {21b}

A data and safety monitoring board (DSMB) will monitor the ongoing trial results and safety data to ensure study integrity, feasibility, and participant well-being. The DSMB will be provided an interim report summarizing only safety information after at least 50 participants have completed follow-up through day 15 (i.e., the end of study allocated treatment).

Subsequent interim analyses will take place in increments of 150 participants, but may be requested by the DSMB more frequently. In these interim analyses, the DSMB will be given the guidance in Table 3 to consider terminating the study for efficacy or inefficacy/harm based on the posterior probabilities for the primary outcome of the odds ratio of the longitudinal proportional odds model:

**Table 3:** Interim monitoring guidance for DSMB with regards to early termination

Probability	Evidence for	Action trigger
*P*_1_ = *P*(OR > 1 | data)	Any benefit	*P*_1_ > 0.95
*P*_2_ = *P*(OR < 1 | data)	Inefficacy or potential harm	*P*_2_ > 0.90

Interim monitoring for futility to detect an odds ratio greater than 1, indicating treatment benefit, was added to the protocol after trial initialization and after 400 participants had been enrolled. The futility monitoring was based on the posterior predictive probability of success (PPoS) of being able to detect the probability of achieving an odds ratio greater than 1 with a posterior probability of at least 90% upon trial completion (i.e., P(OR > 1 | data) > 0.90). This calculation was achieved through upstrapping the remaining number of participants, which resamples with replacement of those who have completed the trial to calculate the posterior probability of success after completing enrollment [17]. The recommendations provided in Table 4 with regards to futility are:

**Table 4:** Futility monitoring guidance for DSMB with regards to termination for futility to detect any significant benefit based on the predictive probability of success

Predictive probability of success (PPoS) action trigger	Action
PPoS < 0.10	Stop for futility
0.10 < PPoS < 0.25	Consider stopping for futility based on trial characteristics (e.g., accrual), secondary endpoints, and external factors
PPoS ≥ 0.25	Continue trial

### Methods for additional analyses (e.g., subgroup analyses) {20b}

The currently recommended approach to subgroup analysis is to prespecify subgroups where there is a clear biological rationale, and otherwise not to create subgroups unless the data indicate differential treatment effects based on potential subgrouping variables. Given how little is known about COVID-19 and possible treatment responses, we will examine the potential of differential treatment effects in key subgroups of interest based upon definitions from the statistical analysis plan (see Additional Document 2) unless otherwise noted:

- Age
- Sex
- Race/ethnicity
- BMI
- Baseline renal function defined as known severe chronic kidney disease requiring dialysis, kidney disease not requiring dialysis, no known history of kidney disease
- Hypertension
- Diabetes defined as diabetes with complications, diabetes without complications, not diabetic
- Cardiovascular disease
- Duration of respiratory symptoms prior to randomization

Interactions will not be tested together within the same model but will be tested one by one. Potentially significant subgroups will be identified by testing the interaction between the covariate and treatment allocation. If an interaction has a posterior probability that *P*(OR>1)> 0.80 or *P*(OR<1)> 0.80, we will proceed with fitting the model within subgroups. For continuous variables, graphical methods will be used to show how the treatment effect changes over the range of the continuous variable.

### Methods in analysis to handle protocol non-adherence and any statistical methods to handle missing data {20c}

The primary analysis is based on intention-to-treat principles, where all patients who have confirmation of receipt of their blinded allocated study drug will be included in their randomized group. This excludes any participants who may be randomized but never received their study drug (e.g., due to logistical issues or for dropping out prior to receipt of study drug).

The safety population will be analyzed similarly to the intention to treat population, where every participant has a receipt of study medication will be included. In the unlikely case, a participant receives the incorrect study drug, patients will be grouped according to the treatments that they received in any safety analyses. If a participant receives both placebo and active treatment, they will be considered as receiving active treatment.

Missing outcome data for an analysis population will be imputed for the outcomes using multiple imputation when necessary. Data on age, gender, comorbidities, and duration of acute respiratory infection prior to randomization should not be missing as they are collected as part of enrollment; however, imputation procedures will be used in cases of missing values.

### Plans to give access to the full protocol, participant-level data and statistical code {31c}

The full protocol and statistical analysis plan will be made available with the publication of this paper as additional files. Statistical code can be made available upon request from the study team.

## Oversight and monitoring

### Composition of the coordinating center and trial steering committee {5d}

The steering committee is composed of the primary investigators of the trial, co-investigators, and dedicated study personnel. The coordinating center at Vanderbilt University Medical Center facilitates the construction of all study data collection instruments, maintains the secure REDCap electronic database, and ensures data validity.

### Composition of the data monitoring committee, its role, and reporting structure {21a}

The principal role of the DSMB is to assure the safety of participants in the trial. They will regularly monitor data from this trial, review and assess the performance of its operations with respect to:

- Review of adverse events
- Review of every death occurring on study
- Interim results of the study for evidence of efficacy or adverse events
- Possible early termination of the trial because of new external information, early attainment of study objectives, safety concerns, or inadequate performance
- Possible modifications in the clinical trial protocol
- Performance of individual centers

The DSMB will consist of three members with expertise in acute infections, acute lung injury, emergency medicine, biostatistics, ethics, and clinical trials. Appointment of all members is contingent upon the absence of any conflicts of interest. All the members of the DSMB are voting members. The unblinded statistician will be responsible for the preparation of all DSMB and adverse event reports and may review unblinded data. The DSMB will develop a charter and review the protocol and sample consent form during its first meeting, with subsequent DSMB meetings scheduled in accordance with the DSMB Charter. Recommendations to end, modify, or continue the trial will be prepared by the DSMB executive secretary. Recommendations for major changes, such as stopping the trial, will be communicated immediately. Other recommendations will be distributed in writing to the coordinator center and institutional review board, which will distribute with instructions for reporting to local IRBs when appropriate.

### Adverse event reporting and harms {22}

Assuring patient safety is an essential component of the study protocol. Lopinavir/ritonavir is approved by the Food and Drug Administration and has been used in clinical practice for decades with a well-established safety profile. The use of lopinavir/ritonavir for the treatment of acute respiratory infection due to COVID-19, however, raises unique safety considerations. This protocol addresses these considerations through:

1. Exclusion criteria designed to prevent the enrollment of patients likely to experience adverse events with receipt of lopinavir/ritonavir
2. On-study monitoring of co-interventions (e.g., newly prescribed medications) and patient characteristics (e.g., healthcare utilization, clinically obtained electrocardiogram) to intervene before adverse events occur
3. Systematic collection of safety outcomes relevant to the use of lopinavir/ritonavir in this setting
4. Structured reporting of adverse events

Each participating investigator has primary responsibility for the safety of the individual participants under his or her care. The investigators will determine daily if any adverse events occur during the period from enrollment through 48 h after completion of the study drug administration and will determine if such adverse events are reportable. Thereafter, adverse events are not required to be reported to the IRB unless the investigator feels the adverse event was related to the study drug or study procedures. Additional details on the classification and reporting of adverse events can be found in the protocol (see Additional Document X).

### Frequency and plans for auditing trial conduct {23}

All records are stored in REDCap, which maintains a system to audit changes and access to the database. Internal auditing will be conducted by study investigators and coordinators and data audits/queries will be conducted by the trial coordinating center prior to locking the database.

### Plans for communicating important protocol amendments to relevant parties (e.g. trial participants, ethical committees) {25}

Protocol amendments will be communicated via email to study investigators and coordinators with additional training (synchronous or asynchronous) arranged as needed. These modifications will also be shared with the trial registry, the single IRB, and trial sponsors.

### Dissemination plans {31a}

The primary paper will be published in a peer-reviewed journal and may also be presented at academic conferences.

## Discussion

While much progress has been made in understanding and addressing the pandemic crisis caused by COVID-19, much work remains to be done. As stated earlier, there is still a need to identify additional outpatient therapies to reduce the progression to severe cases of disease. The TREAT NOW platform seeks to reduce this gap in knowledge by identifying if lopinavir/ritonavir is both effective at reducing progression while maintaining an adequate safety profile.

The dynamic nature of COVID-19 leads to a need for flexibility that has not always been a presence in randomized clinical trials. At the time of the writing of this article, the TREAT NOW platform is hosting a two-arm study, but at the start of the trial, a third arm with the intervention hydroxychloroquine was present before being dropped due to accumulating external evidence (10-14). The advantage of the TREAT NOW platform is the ability to flexibly add new agents in a prospective manner to the platform as the pandemic continues to evolve and new evidence emerges.

There are various strengths and novel elements of TREAT NOW that warrant further discussion. First, in order to minimize face-to-face contact, a traditional prerequisite for participation in many clinical trials, we have developed and implemented a decentralized trial with a robust electronic system to capture baseline data, daily assessment of participants, safety monitoring, and trial endpoints. In addition, the consent process can be completed via electronic means, and no additional specimens are collected that would expose study staff or other individuals to infectious individuals. Accordingly, recruitment can also occur nationally and remotely from enrolling centers. The use of a Bayesian approach is facilitating the use of newer longitudinal proportional odds models that have not previously been derived in a frequentist context. This is advantageous given early guidance from the World Health Organization to use an ordinal outcomes scale, which can capture more granular information than a simple binary outcome, but has statistical challenges for appropriate analysis and interpretation with previously limited means of accounting for rich longitudinal data, such as our daily assessments.

## Trial status

The overall platform is in progress with recruitment having opened on June 1, 2020. The hydroxychloroquine arm was dropped on June 23, 2020. Enrollment into the lopinavir/ritonavir arm ceased on December 17, 2021, with data collection continuing through the first half of the calendar year 2022. The current version of the protocol at the time of writing is version 1.6 (January 26, 2021). There are active discussions on adding new arms to the platform for TREAT NOW.

## Supplementary Information

**Additional file 1.** Study Protocol.

**Additional file 2.** Statistical Analysis Plan.

## Abbreviations

COVID-19: Coronavirus disease 2019
EKG: Electrocardiogram
ICU: Intensive care unit
QTc: QT interval corrected for heart rate
RT-PCR: Reverse transcription-polymerase chain reaction
SARS-CoV-2: Severe acute respiratory syndrome coronavirus 2
WHO: World Health Organization
