# Supplementary material for: Trial of Early Antiviral Therapies during Non-hospitalized Outpatient Window (TREAT NOW) for COVID-19: a summary of the protocol and analysis plan for a decentralized randomized controlled trial
Source: Trials. 2022 Apr 8;23:273. doi: 10.1186/s13063-022-06213-z (PMC8990452; doi:10.1186/s13063-022-06213-z)
Supplement: Supplementary file 1 — Additional file 1. Study Protocol. [file 13063_2022_6213_MOESM1_ESM.pdf]

Trial of Early Antiviral Therapies during Non-hospitalized Outpatient Window (TREAT NOW) for COVID-19

Funding: US Department of Defense and AbbVie

Network: Influenza and other Viruses in the Acutely Ill (IVY) Network

Protocol: Version 1.6

Date: January 26, 2021

TREAT NOW Trial Leadership

Principal Investigators

Adit A. Ginde, MD, MPH
University of Colorado School of Medicine
Email: adit.ginde@cuanschutz.edu

Nathan I. Shapiro, MD, MPH
Beth Israel Deaconess Medical Center
nshapiro@bidmc.harvard.edu

Lead Biostatistician

Alex Kaizer, PhD
Colorado School of Public Health
alex.kaizer@cuanschutz.edu

Data Coordinating Center at Vanderbilt University Medical Center

Gordon Bernard, MD<br>Chris Lindsell, PhD<br>Frank Harrell, PhD	Todd Rice, MD<br>Wesley H. Self, MD, MPH

Signature of Clinical Study Protocol

This signatory agrees to the content of the final clinical study protocol as presented.

Name: Todd Rice, M.D., MSc   Data Coordinating Center Principal Investigator

Date: ____________________________   Signature: ____

Name: Adit A. Ginde, M.D., M.P.H.   Principal Investigator

Date: ____________________________   Signature: ____

Name: Nathan I. Shapiro, M.D., M.P.H.   Principal Investigator

Date: ____________________________   Signature: ____

Table of Contents

Signature of Clinical Study Protocol **3**

ABBREVIATIONS 7

1. STUDY SUMMARY 9

2. TRIAL DESCRIPTION 13

2.1 Background **13**

2.1.1 COVID-19 Infection 13

2.1.2 Promising Candidate Therapies for COVD-19 13

2.1.3 Rationale for a Randomized Trial Early Treatment among Outpatients 14

2.2 Study Aims **14**

2.2.1 Study aim 14

2.2.2 Study hypothesis 14

2.3 Study Design **15**

3. STUDY POPULATION AND ENROLLMENT 15

3.1 Inclusion Criteria **15**

3.2 Exclusion Criteria **15**

3.3 Justification of Exclusion Criteria **16**

3.4 Screening **16**

3.5 Assessment of Eligibility and Exclusion Tracking **17**

3.6 Process of Obtaining Informed Consent **17**

3.7 Randomization and Blinding **18**

4. STUDY INTERVENTIONS 18

4.1 Treatment of Study Participants **18**

4.2 Lopinavir/Ritonavir Group **19**

4.3 Control Group **20**

4.4 Co-Interventions **20**

4.5 On-Study Monitoring **20**

4.6 Criteria for Stopping Study Drug **21**

5. OUTCOMES 21

5.1 Primary Outcome **21**

5.2 Secondary Outcomes **22**

5.3 Safety outcomes **22**

5.4 Rationale for Primary Outcome **22**

6. DATA COLLECTION 23

6.1 Baseline Variable Collection **24**

6.2 Daily Assessments between Randomization and Study Day 16, and then at Day 29 .... **24**

7. STATISTICAL CONSIDERATIONS 25

8. DATA QUALITY MONITORING AND STORAGE 26

8.1 Data Quality Monitoring **26**

8.2 Data Storage **26**

9. RISK ASSESSMENT 26

9.1 Potential Risk to Participants **26**

9.1.2 Potential risks of receiving lopinavir/ritonavir 26

9.1.3 Potential risks of receiving placebo with COVID-19 27

9.2 Minimization of Risk **27**

9.3 Potential Benefit **28**

9.4 Risk in Relation to Anticipated Benefit **28**

10. HUMAN SUBJECTS PROTECTIONS 28

10.1 Selection of Subjects **28**

10.2 Informed Consent **28**

10.3 Withdrawal of Consent **29**

10.4 Confidentiality **29**

11. ADVERSE EVENTS 29

11.1 Adverse Event Definitions **30**

11.2 Safety Monitoring **30**

11.3 Serious Adverse Events **31**

12. Data and Safety Monitoring Board (DSMB) 32

13. APPENDICES 33

Appendix A. Schedule of Events **33**

Appendix B. Potential medication interactions with lopinavir/ritonavir **34**

Appendix C: Adverse Event Reporting and Unanticipated Events **35**

C.1. Unanticipated Problems (UP) 35

C.2. Determining Relationship of Adverse Events to Study Drug or Study Procedures 35

C.3. Clinical Outcomes that may be Exempt from Adverse Event Reporting 36

C.4. Decision tree for determining if an adverse event is reportable 37

14. REFERENCES 38

ABBREVIATIONS

ACE	Angiotensin-converting enzyme
AE	Adverse event
ALT	Alanine aminotransferase
ARDS	Acute respiratory distress syndrome
AST	Aspartate aminotransferase
AUC	Area under the curve
BAL	Bronchoalveolar lavage
BID	twice daily
CFR	Code of Federal Regulations
COVID-19	Coronavirus Disease 2019
CYP3A	Cytochrome P4503A
DCC	Data Coordinating Center
DSMB	Data safety monitoring board
ECMO	Extracorporeal membrane oxygenation
eCRF	Electronic case report forms
EKG	Electrocardiogram
EUA	Emergency Use Authorization
FDA	Food & Drug Administration
HIPAA	Health Insurance Portability and Accountability Act
HIV	Human immunodeficiency virus
HR	Hazard ratio
IC_{50}	Half maximal inhibitory concentration
ICU	Intensive care unit
ID	Identification
IL1	Interleukin-1
IL6	Interleukin-6
IP	Investigational product
IRB	Institutional Review Board
IRIS	Immune reconstitution inflammatory syndrome
IVY Network	Influenza Vaccine Effectiveness in the Critically Ill Network
KDIGO	Kidney Disease Improving Global Outcomes
LFT	Liver function test
LPV/r	Lopinavir/ritonavir
MERS-CoV	Middle East respiratory syndrome coronavirus 2
NHLBI	National Heart, Lung, and Blood Institute

NIH	National Institutes of Health
PI	Principal investigator (a clinician responsible for one site)
PK/PD	Pharmacokinetics/pharmacodynamics
PPE	Personal protective equipment
QD	once daily
QR code	Quick response code
QTc	QT interval corrected for heart rate
REDCap	Research Electronic Data Capture
RT-PCR	Reverse transcription polymerase chain reaction
SAE	Serious adverse events
SARS-CoV-2	Severe acute respiratory syndrome coronavirus 2
sIRB	Single IRB
SUSAR	Suspected unexpected serious adverse reaction
US	United States
VCC	Vanderbilt Coordinating Center
VAS	Visual Analog Scale

1. STUDY SUMMARY

**Title**	Trial of Early Antiviral Therapies during Non-hospitalized Outpatient Window
**Acronym**	TREAT NOW
**Background**	Effective therapies for COVID-19 are urgently needed. **Lopinavir/Ritonavir** (Kaletra) is an antiviral agent used to treat HIV-1 and is a potent *in vitro* inhibitor of SARS-CoV-2, the virus that causes COVID-19. Initial clinical data have suggested possible relevance as a potential therapeutic agent early in the course of patients with COVID-19.
**Study Design**	Blinded, multicenter, placebo-controlled randomized clinical trial
**Intervention Group**	Lopinavir/ritonavir 400 mg/100 mg orally twice daily for twenty-eight doses (Days 1-14)
**Control Group**	Placebo (unmatched) orally twice daily for 14 days
**Sample Size**	Up to 600 patients
**Inclusion Criteria**	1. Age ≥18 years<br>2. Laboratory-confirmed SARS-CoV-2 infection by RT-PCR or other molecular test, or by antigen test with emergency use authorization or full approval and collected within the past 6 days<br>3. Current symptoms of acute respiratory infection for ≤6 days, defined as one or more of the following:<br>o cough<br>o fever<br>o shortness of breath<br>o chest pain<br>o abdominal pain<br>o nausea/vomiting<br>o diarrhea<br>o body aches<br>o weakness/fatigue
**Exclusion Criteria**	1. Prisoner<br>2. Pregnancy<br>3. Breast feeding<br>4. Two individuals from the same household are not enrolled in the study<br>5. Unable to randomize within 6 days after onset of acute respiratory infection symptoms<br>6. Hospitalization within the 6 days prior to randomization<br>7. Inability to swallow oral medications<br>8. Refusal or inability to be contacted and participate in daily symptom/safety monitoring in English or Spanish during the two-week follow-up period<br>9. Previous enrollment in this trial<br>10. Known severe chronic kidney disease requiring dialysis

	11. Known liver disease (cirrhosis or >3 times upper limit of normal for AST or ALT in medical record if available)<br>12. Known hepatitis B or hepatitis C infection<br>13. Known history of jaundice<br>14. Current heavy alcohol use, defined as 8 drinks or more per week for women or 15 drinks or more per week for men<br>15. Known seizure disorder<br>16. Known HIV infection<br>17. Known history of pancreatitis<br>18. Known history of prolonged QT interval (Long QT Syndrome, patient report, or QTc >500 milliseconds on most recently available electrocardiogram within the past 2 years)<br>19. Receipt of >1 dose of lopinavir/ritonavir in the 10 days prior to enrollment<br>20. Known allergy to lopinavir/ritonavir<br>21. Currently prescribed (with planned continuation) or planned administration during 14-day study period of medication at high risk for QT prolongation as follows:<br>*Antiarrhythmics:* Amiodarone, disopyramide, dofetilide, dronedarone, flecainide, ibutilide, procainamide, propafenone, quinidine, sotalol<br>*Anti-cancer:* Arsenic trioxide, oxaliplatin, vandetanib<br>*Antidepressants:* Amitriptyline, citalopram, escitalopram, imipramine<br>*Antimicrobials:* azithromycin, ciprofloxacin, clarithromycin, erythromycin, fluconazole, levofloxacin, moxifloxacin, pentamidine, hydroxychloroquine<br>*Antipsychotics:* aloperidol, chlorpromazine, droperidol, olanzapine, pimozide, quetiapine, thioridazine, risperidone, ziprasidone<br>*Others:* cilostazol, cimetidine, cisapride, donepezil, methadone, ondansetron, sumatriptan<br>22. Currently prescribed (with planned continuation) or planned administration during 14-day study period of any of the following medications: alfuzosin, apalutamide, astemizole, ergot-containing medicines (including dihydroergotamine mesylate, ergotamine tartrate, methylergonovine), lomitapide, lovastatin, lurasidone, midazolam, phenobarbital, phenytoin, ranolazine, rifampin, sildenafil, simvastatin, rivaroxaban, St. John's Wort, terfenadine, triazolam. Patients who are on warfarin or fluticasone will be advised to contact their primary care provider to advise them that they are in the trial and possibly receiving lopinavir/ritonavir which can influence levels of either drug and may require more frequent monitoring.
**Randomization**	Eligible patients will be randomized through a central electronic system in a 1:1 ratio, to lopinavir/ritonavir (intervention) versus placebo (control). Randomization will be stratified by site and age (≥65 years or <65 years).
**Blinding**	Patients, treating clinicians, trial personnel, and outcome assessors will be blinded to group assignment.
**Primary Outcome**	Modified COVID Ordinal Outcomes Scale to Study Day 15:<br>1. Death

	2. Hospitalized on mechanical ventilation or ECMO<br>3. Hospitalized on supplemental oxygen<br>4. Hospitalized not on supplemental oxygen<br>5. Not hospitalized with symptoms and limitation in activity<br>6. Not hospitalized with symptoms but with no limitation in activity<br>7. Not hospitalized without symptoms nor limitation in activity
**Secondary Outcomes**	• Modified COVID Ordinal Outcome Scale to Study Day 8<br>• Modified COVID Ordinal Outcome Scale to Study Day 29<br>• Proportion of patients hospitalized to Study Day 29<br>• Time to hospitalization to Study Day 29<br>• Time to symptom resolution to Study Day 29<br>• All-cause, all-location mortality to Study Day 29<br>• Oxygen-free days through Study Day 29<br>• Fever-free days to Study Day 29<br>• Ventilator-free days through Study Day 29<br>• Vasopressor-free days through Study Day 29<br>• ICU-free days through Study Day 29<br>• Hospital-free days through Study Day 29
**Safety Outcomes**	• Seizure<br>• Atrial or ventricular arrhythmia<br>• Cardiac arrest<br>• *Elevation in aspartate aminotransferase or alanine aminotransferase to twice the local upper limit of normal AND at least doubling over known baseline<br>• *Acute pancreatitis<br>• *Acute kidney injury by KDIGO criteria<br>• Receipt of renal replacement therapy<br>• *Symptomatic hypoglycemia<br>• *Anemia or thrombocytopenia<br>• Severe dermatologic reaction<br><br>*For participants whose symptoms are significant enough to trigger a clinical work-up and thus have clinically available testing
**Analysis**	Using intention-to-treat principles, we will compare the primary outcome (Modified COVID Ordinal Outcomes Scale score on Study Day 15) between patients the intervention group (lopinavir/ritonavir) and the control group using a proportional odds model, adjusted for the following co-variables: age, gender, and duration of acute respiratory infection symptoms prior to randomization. With type I error of 0.05, enrolling 540 patients in the primary population will provide 90% power to detect an odds ratio of 1.75. Accounting for a 10% loss to follow-up rate, we will plan to enroll **600 patients**. This sample size and power assumes a frequentist analysis, which is generally more conservative than what is needed for a Bayesian approach. We will plan an initial DSMB evaluation for

	safety and feasibility after n=100 patients have been enrolled; efficacy will not be considered at this analysis. The trial will then have frequent interim analyses with details on specifics on timing of meetings, as well as efficacy and futility stopping rules to be detailed in the Statistical Analysis Plan, to be finalized prior to the n=100 interim analyses.

2. TRIAL DESCRIPTION

2.1 Background

Coronavirus Disease 2019 (COVID-19) is an acute respiratory infectious illness caused by *severe acute respiratory syndrome coronavirus 2* (SARS-CoV-2).[1,2] Although the epidemiology is not fully elucidated, most adults with COVID-19 appear to experience fever, cough, and fatigue and then recover within 1-3 weeks. However, a portion of adults with COVID-19 develop severe illness, typically manifesting as pneumonia and hypoxemic respiratory failure, with continued progression to acute respiratory distress syndrome (ARDS) and death in some cases.[1–3] There is an urgent need for outpatient therapies with a demonstrated ability to improve recovery progression of COVID-19 to severe illness. Based on mechanism of action and early clinical experiences, several agents currently available in the U.S. are proposed as potential therapies to prevent disease progression.[4–6] Among these potential therapies, lopinavir/ritonavir has generated substantial interest due to antiviral and immunomodulatory activity and established safety profiles with FDA approval for use in other conditions. In this trial, we will evaluate effectiveness and safety of lopinavir/ritonavir for the early treatment of adults with COVID-19 in the outpatient setting, prior to hospitalization.

2.1.1 COVID-19 Infection

COVID-19 was first identified as a cluster of cases of pneumonia among a group of workers from a seafood wholesale market in Wuhan, China in November 2019.[7] This observation, along with subsequent viral genotyping showing significant genetic similarities to the bat coronaviruses[8] suggest a zoonotic origin, although the specific reservoir and intermediary species remain unclear.[9] The COVID-19 infection represents the seventh coronavirus known to cause disease in humans.[10] Four of the coronaviruses viruses are known to cause symptoms of the common cold in immunocompetent individuals while two others (SARS-CoV and MERS-CoV) have caused recent outbreaks of severe and sometimes fatal respiratory diseases.[11] SARS-CoV-2 appears to exploit the same cellular receptor as SARS-CoV and MERS-CoV,[12] and its severity may similarly result from a predilection for intrapulmonary epithelial cells over cells of the upper airways.[13,14]

Since the first documented human case, COVID-19 has spread exponentially with 1,447,412 confirmed cases and 93,425 deaths worldwide as of April 9, 2020. While most patients recover after a mild, brief illness with fever and cough, the disease has a clinical spectrum ranging from asymptomatic infection[15] to ARDS and death.[16] The most common reasons for ICU care are respiratory failure and ARDS, with a minority developing shock and possibly cardiomyopathy.[17] The case fatality rate is estimated to be 0.25% to 3.0%.[18]

2.1.2 Promising Candidate Therapies for COVD-19

Several agents currently available in the US are proposed as potential therapies to halt disease progression.[4-6]

Lopinavir/ritonavir (Kaletra), an antiretroviral medication used to treat HIV-1, also has potent antiviral activity and promising early clinical data against coronaviruses, including those causing SARS and MERS.[22–26] Lopinavir is a HIV type 1 aspartate protease inhibitor; when combined with ritonavir, the plasma half-life of lopinavir is increased. Lopinavir was first identified as a potential therapy for severe

coronaviruses as demonstrating strong *in vitro* inhibitory activity against both SARS-CoV, the virus which causes Severe Acute Respiratory Syndrome (SARS), and MERS-CoV, the virus which causes Middle East Respiratory Syndrome (MERS).[27,28] Prior open label studies of lopinavir-ritonavir suggested the potential to improve clinical outcomes and decrease viral load in patients with SARS and MERS.[29–32]

A recent randomized open-label trial of lopinavir/ritonavir in hospitalized patients with COVID-19 failed to demonstrate a treatment benefit.[3] However, the trial was underpowered for clinically important endpoints and enrolled patients at an advanced stage of disease, a median of 13 days after symptom onset, when antiviral activity is likely much less important. Still, the observed mortality in the lopinavir/ritonavir group was 5.8% lower than the control group (95% confidence interval, -17.3 to 5.7), as well as an observed faster time to clinical improvement that was also not statistically significant (HR 1.24, 95%CI 0.90-1.72). Accordingly, substantial enthusiasm remains for early treatment of COVID-19, particularly prior to hospitalization.

2.1.3 Rationale for a Randomized Trial Early Treatment among Outpatients

Initial COVID-19 symptoms develop approximately 2-10 days after infection with the SARS-CoV-2 virus.[33–35] Most adults recover without complications, but hospitalized patients typically experience pneumonia leading to acute hypoxemic respiratory compromise/failure, and in some cases, ARDS and death. The first 4-8 days of illness typically manifest with conditions consistent with a viral syndrome such as fevers and cough. Those experiencing a milder course typically recover after this initial period; those on a more severe course will progress to respiratory failure and ARDS, which usually occurs 7-12 days after the onset of symptoms.[36,37]

The period between onset of symptoms and the development of hypoxemia requiring hospitalization is a critical therapeutic window for treatment to halt disease progression. Early data suggest that mortality rates are highest in the elderly, immunocompromised, and those with chronic cardiopulmonary disease. However, young adults remain at high risk of complications, as younger adults (age 20-44 years) comprise approximately 25% of COVID-19-hospitalized patients.

COVID-19 is an unprecedented public health crisis, and there is significant interest in finding effective therapies, specifically in repurposing approved medications with widespread availability and known safety profiles. Efficacy and safety data for lopinavir/ritonavir from randomized trials are critical to provide evidence-based therapy for the ongoing COVID-19 pandemic. Our proposed trial will efficiently assess the potential candidate therapy and provide a structure for rapid integration of other candidate drugs in future clinical trials.

2.2 Study Aims

2.2.1 Study aim

To determine the effectiveness and safety of early treatment with lopinavir/ritonavir versus placebo in outpatient adults with COVID-19.

2.2.2 Study hypothesis

Early initiation of lopinavir/ritonavir will reduce disease progression and improve clinical outcomes among outpatient adults with COVID-19.

2.3 Study Design

We will conduct an investigator-initiated, multicenter, blinded, placebo-controlled, randomized clinical trial evaluating lopinavir/ritonavir vs placebo for early treatment of adults with COVID-19 in the outpatient setting prior to hospitalization. Patients, treating clinicians, and study personnel will all be blinded to study group assignment.

3. STUDY POPULATION AND ENROLLMENT

3.1 Inclusion Criteria

1. Age ≥18 years
2. Laboratory-confirmed SARS-CoV-2 by RT-PCR or other molecular test, or by antigen test with FDA emergency use authorization or full approval and collected within the past 6 days
3. Current symptoms of acute respiratory infection for ≤6 days, defined as one or more of the following:
    - cough
    - fever
    - shortness of breath
    - chest pain
    - abdominal pain
    - nausea/vomiting
    - diarrhea
    - body aches
    - weakness/fatigue

3.2 Exclusion Criteria

1. Prisoner
2. Pregnancy
3. Breast feeding
4. Two individuals from the same household are not enrolled in the study
5. Unable to randomize within 6 days after onset of acute respiratory infection symptoms
6. Hospitalization within the 6 days prior to randomization
7. Inability to swallow oral medications
8. Refusal or inability to be contacted and participate in daily symptom/safety monitoring in English or Spanish during the two-week follow-up period
9. Previous enrollment in this trial
10. Known severe chronic kidney disease requiring dialysis
11. Known liver disease (cirrhosis or >3 times upper limit of normal for AST or ALT in medical record if available)
12. Known hepatitis B or hepatitis C infection
13. Known history of jaundice
14. Current heavy alcohol use, defined as 8 drinks or more per week for women or 15 drinks or more per week for men
15. Known seizure disorder

16. Known HIV infection
17. Known history of pancreatitis
18. Known history of prolonged QT interval (Long QT Syndrome, patient report, or QTc >500 milliseconds on most recently available electrocardiogram within the past 2 years)
19. Receipt of >1 dose of hydroxychloroquine, chloroquine, or lopinavir/ritonavir in the 10 days prior to enrollment
20. Known allergy to lopinavir/ritonavir
21. Currently prescribed (with planned continuation) or planned administration during 14-day study period of medication at high risk for QT prolongation as follows:
    Antiarrhythmics: Amiodarone, disopyramide, dofetilide, dronedarone, flecainide, ibutilide, procainamide, propafenone, quinidine, sotalol
    Anti-cancer: Arsenic trioxide, oxaliplatin, vandetanib
    Antidepressants: Amitriptyline, citalopram, escitalopram, imipramine
    Antimicrobials: azithromycin, ciprofloxacin, clarithromycin, erythromycin, fluconazole, levofloxacin, moxifloxacin, pentamidine, hydroxychloroquine
    Antipsychotics: aloperidol, chlorpromazine, droperidol, olanzapine, pimozide, quetiapine, thioridazine, risperidone, ziprasidone
    Others: cilostazol, cimetidine, cisapride, donepezil, methadone, ondansetron, sumatriptan
22. Currently prescribed (with planned continuation) or planned administration during 14-day study period of any of the following medications: alfuzosin, apalutamide, astemizole, ergot-containing medicines (including dihydroergotamine mesylate, ergotamine tartrate, methylergonovine), lomitapide, lovastatin, lurasidone, midazolam, phenobarbital, phenytoin, ranolazine, rifampin, sildenafil, simvastatin, rivaroxaban, St. John's Wort, terfenadine, triazolam. Patients who are on warfarin or fluticasone will be advised to contact their primary care provider to advise them that they are in the trial and possibly receiving lopinavir/ritonavir which can influence levels of either drug and may require more frequent monitoring.

3.3 Justification of Exclusion Criteria

Since this is intended as an outpatient trial, patients hospitalized within 6 days prior to enrollment/randomization are excluded. The other exclusion criteria are primarily designed for patient safety. In addition to excluding specific vulnerable populations (e.g., prisoners), these criteria are designed to exclude patients for whom receipt of lopinavir/ritonavir might increase the risk of serious adverse events. Lopinavir/Ritonavir has been routinely prescribed to outpatients with HIV-1 without extensive cardiac workups, laboratory testing, or monitoring. In addition, we have incorporated guidelines for safe outpatient prescribing of lopinavir/ritonavir without a baseline electrocardiogram or outpatient cardiac monitoring[40] and have put into place a method of monitoring newly prescribed medications during the 14-day intervention period.

3.4 Screening

The site investigator or delegate will screen for non-hospitalized patients with laboratory confirmed COVID-19 (that is, a positive laboratory test for SARS-CoV-2 by RT-PCR or other molecular method, or by antigen test with FDA approval or emergency use authorization and collected within the past 6 days). The source of these patients will be primarily patients seen and sent home from the enrolling hospital

emergency department, urgent care, primary care, virtual care/telemedicine visits, or testing centers. We may also advertise the study at outpatient testing sites and more broadly for self-referral to the study.

3.5 Assessment of Eligibility and Exclusion Tracking

For patients who appear to meet eligibility criteria after screening, we will complete an electronic case report form to determine eligibility and track exclusions. We will access and store the electronic case report form in the electronic database. At the time of entry into the screening database, we will assign the patient a screening number.

If a patient appears to meet all eligibility criteria, the site investigator or delegate will approach the patient to confirm eligibility, discuss potential study recruitment, and proceed with informed consent. Most patients will no longer be in the healthcare setting and therefore we anticipate that this discussion will occur primarily by telephone or videophone to the patient's home (test pending patients are typically advised by their clinicians to home quarantine while symptomatic and test results are pending). We may advertise the study at both the testing sites as well as more broadly.

For all excluded patients, including refusal by the patient to participate, we will collect a small number of variables, including month and year patient met screening criteria and reason(s) patient was excluded to allow for reporting in the CONSORT diagram of the manuscript. Due to the nature of this trial in the outpatient setting and for staff safety and personal protective equipment (PPE) conservation, these encounters will usually occur via telephone or videophone.

3.6 Process of Obtaining Informed Consent

We will obtain written informed consent from the patient. We will not enroll patients who lack decision-making capacity due to logistical issues with remote study medication administration, safety monitoring, and accurate data collection.

In-person visits for patients with known COVID-19 who are deemed stable for outpatient management and home quarantine would violate infection control principles and policies. Given the infectious risk from COVID-19 and potential shortages of personal protective equipment (PPE), there is a moral and practical imperative to minimize face-to-face contact between patients and non-clinical personnel. Therefore, we will use "no-touch" consent procedures for this trial, employing an electronic remote consent process to obtain written informed consent.

Electronic approach

1. A link for the electronic consent is sent to the subject.
2. Research staff contact the patient by telephone or videophone (method dictated by institutional policy) to have an informed consent conversation. *This step confirms subject identity*.
3. If they consent, the patient signs the consent form. This can be:
    a. an actual signature (often tracing their finger on the screen) OR
    b. a username and password specific to the individual signing

This approach complies with relevant regulations and sub-regulator guidance at 45 CFR 46.117, 45 CFR 164.512, 21 CFR 11 Subpart C (11.100–11.300), https://www.hhs.gov/ohrp/regulations-and-

policy/guidance/use-electronic-informed-consent-questions-and-answers/index.html,
https://www.fda.gov/regulatory-information/search-fda-guidance-documents/informed-consent

We will provide the information for the informed consent discussion in a formal document that has been approved by the IRB and in a language comprehensible to the potential participant, using an interpreter if necessary. Currently, English and Spanish documents are approved for use in this trial. The information presented in the consent form and by the research staff will detail the nature of the trial and what is expected of participants, including any potential risks or benefits of taking part. We will clearly state that the participant is free to withdraw from the trial at any time for any reason without prejudice to future care, and with no obligation to give the reason for withdrawal. Where a patient does not speak English, a translated Spanish consent and qualified interpreter will be employed, using similar "no-touch" principles. Use of a telephone or video interpreter and the interpreter's identity will be documented on the electronic consent.

After allowing the potential participant time to read the informed consent document, research staff will answer any additional questions.

3.7 Randomization and Blinding

We will randomize eligible participants through a central electronic system in a 1:1 ratio to lopinavir/ritonavir (intervention) versus placebo (control). A randomized group assignment is provided to the site investigator or delegate from a centralized, web-based platform. Randomization will require provision of the screening number and confirmation of patient eligibility. In the future, we may also consider adding an intervention group.

We will perform the randomization in permuted blocks of varying size and stratified by site and age (≥65 years or <65 years). We will store the randomization sequence allocation on a secure server, which will not be available to site study personnel. Each participant will receive a computer-generated study ID number. The computer-generated study ID number and shipping information will be provided to the pharmacy who will provide a dose pack containing lopinavir/ritonavir or placebo.

The participant, treating clinicians, study personnel, and outcome assessors will all remain blinded to group assignment until after the database is locked and blinded analysis is completed. Only Belmar Pharmacy, who is distributing study medication, and one member of the biostatistical team who is preparing closed DSMB interim reports will be unblinded. Specifically, study medication will be dispensed with packaging and labelling that would blind treatment assignment. Unblinding will occur only if required for subject safety or treatment at the request of the treating clinician.

4. STUDY INTERVENTIONS

4.1 Treatment of Study Participants

A summary of the trial's schedule of events is included in Appendix A.

Timing of study procedures is based on the time of randomization, which is defined as "Time 0". Study Day 1 is defined as the day of receipt of the first dose of study drug. We will assess the primary outcome on Study Day 15, which corresponds to 14 days (2 weeks) after the initiation of study drug.

A research physician will prescribe a study medication that a central pharmacy (Belmar Pharmacy) will fill at no charge to the patient and will arrive through overnight mail (typically next day by 10:00AM). The study medication will arrive in blinded blister packs labelled as lopinavir/ritonavir or placebo with the appropriate daily instruction for administration. The goal is initiation of treatment within one day of randomization (Study Day 1).

On Study Days 1-15, the patient will complete brief electronic data collection form through a secure electronic platform that will confirm receipt and administration of study drug and document the number and reason for any missed doses. For those with missing electronic data, telephone follow-up will provide an additional opportunity to clarify study drug administration, as needed. If the participant is hospitalized prior to completion of the study medication, an attempt will be made to continue study medication. However, study medication may be discontinued if felt necessary by the treating clinician in consultation with the local investigator. Unblinding will occur only if required for subject safety or treatment at the request of the treating clinician.

4.2 Lopinavir/Ritonavir Group

Participants assigned to the lopinavir/ritonavir arm will receive lopinavir/ritonavir 400 mg/100 mg orally twice daily for 28 doses (Days 1-14). Medication dose packs will contain all 28 doses labelled by Study Day.

Rationale for Drug Selection of Lopinavir/Ritonavir

We are administering lopinavir/ritonavir (LPR/r) in standard dosing: lopinavir/ritonavir 400 mg/100 mg twice daily for 28 doses (Days 1-14). LPV/r as Kaletra tablets is dosed based upon extensive PK/PD study evaluations in healthy and HIV+ subjects as reported in its US and European labels (400/100mg BID or 800/200mg QD) - see https://www.accessdata.fda.gov/drugsatfda_docs/label/2016/021251s052_021906s046lbl.pdf and https://www.medicines.org.uk/emc/product/221/smpc. The mean in vitro antiviral IC_{50} of lopinavir against HIV in the absence of human serum, is 6.5 nM. The IC_{50} of LPV vs SARS viruses and in particular SARS-CoV-2 is believed to be approximately 1-10μM.[23,25] Comparison of these potencies is limited as data are uncontrolled and assay procedure differ, but it suggests SARS-CoV-2 replication is approximately 100 - 1000-fold less susceptible to LPV/r-mediated replication than HIV. PK/PD comparisons across unrelated viral infections with overtly different clinical manifestations also presents significant translational insight limitations, but the safety/efficacy and PK/PD for LPV/r against HIV is well known and enables a guide to a pragmatic and safety-reasoned dosing regimen. A regimen of 400/100 mg BID should achieve near steady state concentrations as seen in HIV therapy within 3 to 4 days. This should align with an acceptable safety profile coupled with rapid achievement of LPV/r exposure to efficacious levels associated with HIV clinical antiviral activity, and possibly some effect on SARS-C0V-2 replication inhibition in patients. LPV/r dosing BID, or 800/200mg QD in adults enables unbound LPV exposure at significantly higher levels than reported by the HIV in vitro antiviral IC_{50}. Multiple dosing with 400/100 mg LPV/r twice daily for 14 days produces mean ± SD LPV peak plasma concentration (Cmax) of 12.3 ± 5.4 μg/ml. The mean steady-state trough concentration prior to the morning dose was 8.1 ± 5.7 μg/ml. LPV AUC over a 12 hour dosing interval averages 113.2 ± 60.5 μg/h/m. LPV and RTV are 98–99% bound to the blood plasma proteins, which indicates that unbound LPV exposure to be approximately 10 - 100-fold greater than the in vitro HIV IC_{50} value, and within an

order of magnitude as reported for SARS-C0V-2 value, thereby making clinical testing by the regimen described as reasoned-from a potential antiviral perspective. If unbound drug levels are higher inpatient BAL fluid, then a more favourable ratio is potentially achievable.

4.3 Control Group

Participants randomized to the control group will receive placebo orally twice daily for 14 days. Medication dose packs will contain all 28 doses labelled by Study Day.

While patients who receive the lopinavir/ritonavir will not have a matched placebo, the packaging would indicate study medication (lopinavir/ritonavir, or placebo) and participants will not be aware that there is no matched placebo for lopinavir/ritonavir. Thus, even if the participant searches the internet for pill markings, it is entirely possible from the patient perspective that this could be a marched placebo. Exclusion criteria will be exercised to ensure that two individuals from the same household are not enrolled in the study and that telephone assessments during the study avoid discussion of the appearance of the study medication.

4.4 Co-Interventions

This trial will control the use of lopinavir/ritonavir, or placebo during the 14-day intervention period. Enrolled participants will not receive open-label lopinavir/ritonavir during the 14-day intervention period, unless the patient is hospitalized, and the treating clinician wishes to unblind the trial and use these medications open label. The treating clinicians will make all other treatment decisions without influence from the protocol. Administration of other antiviral, immunomodulatory, or other COVID-19 directed novel medications ("rescue therapy") will be allowed. We will record the co-administered COVID-19 directed medications in the case report form. The patient will be allowed to take antipyretics at home as needed.

4.5 On-Study Monitoring

All participants enrolled in the study will be outpatients (not hospitalized) and therefore initial monitoring will be remote as part of routine outpatient clinical care with treating physicians and nurses (we anticipate primarily virtual care/telehealth or routine telephone follow-up). The research staff will provide additional remote monitoring through daily electronic collection of symptoms, healthcare utilization, clinically obtained electrocardiograms, newly prescribed medications, and adverse event reporting with supplemental telephone follow-up as needed. The research team may flag potential issues but will defer specific decisions about additional care and hospital transfer to usual outpatient clinical care. Lopinavir/ritonavir has been used extensively for outpatient treatment of other conditions without the need for additional monitoring during short term therapy.

Between baseline and Study Day 15, study personnel will ascertain and review the initiation of any new medications daily and evaluate for potential medication interactions with lopinavir/ritonavir (see Appendix B). If a medication that is considered to be contraindicated with lopinavir/ritonavir is discovered, site physician investigator or designee will contact the patient to discuss if stopping study drug is appropriate or if the medication in question may be stopped or substituted in consultation with the prescribing/treating clinician. If a medication with a potential interaction with lopinavir/ritonavir is identified, study personnel will contact the treating clinician to ensure they are aware of the potential interaction, as needed. Treating clinicians will determine whether an alternative medication would be

appropriate or whether the risk-benefit ratio favors continuing the medication with the known potential interaction.

4.6 Criteria for Stopping Study Drug

We will stop administration of the blinded study drug temporarily or permanently for (a) adverse events without evidence of an alternative cause to the patient's symptoms, (b) results of on-study monitoring, or (c) clinical deterioration if requested by treating clinician.

If a patient experiences an adverse event that the patient treating clinicians, or investigators feel merits temporarily or permanently stopping the study drug, we will stop the study drug. We will record the explanation for stopping the study drug in the case report form, and we will record and report the adverse event according to the adverse event guidelines below. If the adverse event resolves to the extent that the patient, treating clinicians, and investigators feel that resuming the study drug is appropriate, we will resume the study drug, and we will record this information in the case report form.

If an EKG is obtained as part of routine clinical care after enrollment and the QTc is greater than or equal to 500 ms, we will discontinue the study drug, unless a repeat EKG after at least 24 hours demonstrates the QTc is less than 500 ms, at which time we will direct the patient to resume the study drug. We will record both the value for the QTc and the decision to continue or stop the study drug in the case report form. If the daily on-study monitoring by study personnel for medication interactions indicates a potential interaction with a medication that treating clinicians feel is required for the optimal treatment of the patient and with which treating clinicians and the investigator feel it would be unsafe to administer lopinavir/ritonavir, we will stop study drug and record the reason in the case report form.

For patients who experience clinical deterioration requiring hospitalization or toxicity potentially related to study drug, for which treating clinicians feel optimal care would include stopping the study drug and unblinding group assignment, we will stop the study drug, and the site investigator will contact the coordinating center to receive the unblinded study group assignment. We will defer any additional treatments to the treating clinicians. In this situation, we will record the following data in the case report form: the criteria met for clinical deterioration; the reason for stopping study drug and unblinding; use of other antivirals, and immunomodulators; and study outcomes. Crossovers from placebo to lopinavir/ritonavir will be recorded and reported to the DSMB at DSMB reviews and interim analyses. The primary analysis will be intention-to-treat.

5. OUTCOMES

5.1 Primary Outcome

The **primary outcome** is clinical status on the Modified COVID Ordinal Outcomes Scale to Day 15, incorporating more granular description of symptoms at the milder end of the scale for this outpatient trial. In addition, we collapsed non-invasive ventilation with mechanical ventilation and high flow nasal oxygen with supplemental oxygen to improve pragmatism of in-hospital data collection (source data may come from the patient or alternate contact if hospital records are not accessible). This outcome is in use by COVID-19 trials globally, including those conducted by the World Health Organization, and will facilitate pooling and comparison of data across trials.

1. Death

2. Hospitalized on mechanical ventilation or ECMO
3. Hospitalized on supplemental oxygen
4. Hospitalized not on supplemental oxygen
5. Not hospitalized with symptoms and limitation in activity
6. Not hospitalized with symptoms but with no limitation in activity
7. Not hospitalized without symptoms or limitation in activity

5.2 Secondary Outcomes

- Modified COVID Ordinal Outcome Scale at Study Day 8
- Modified COVID Ordinal Outcome Scale at Study Day 29
- Proportion of patients hospitalized to Study Day 29
- Time to hospitalization to Study Day 29
- Time to symptom resolution to Study Day 29
- All-cause, all-location mortality to Study Day 29
- Oxygen-free days through Study Day 29
- Fever-free days to Study Day 29
- Ventilator-free days through Study Day 29
- Vasopressor-free days through Study Day 29
- ICU-free days through Study Day 29
- Hospital-free days through Study Day 29

5.3 Safety outcomes

- Seizure
- Atrial or ventricular arrhythmia
- Cardiac arrest
- *Elevation in aspartate aminotransferase or alanine aminotransferase to twice the local upper limit of normal, AND at least doubling over known baseline
- *Acute pancreatitis
- *Acute kidney injury by KDIGO criteria
- Receipt of renal replacement therapy
- *Symptomatic hypoglycemia
- *Anemia or thrombocytopenia
- *Severe dermatologic reaction

*For participants whose symptoms are significant enough to trigger a clinical work-up and thus have clinically available testing and diagnoses:

5.4 Rationale for Primary Outcome

COVID-19 has a broad spectrum of clinical severity. Among non-hospitalized patients, most recover without experiencing critical illness.[42] Designing a trial with statistical power to detect a meaningful difference in hospital-free days, ICU-free days, or mortality might require an unfeasibly large sample size and could miss significant morbidity experienced by outpatients with COVID-19. Since the majority of

morbidity from COVID-19 relates to hypoxemia, the fact that this outcome is tied to degree of hypoxemic respiratory failure increases its face validity and relevance. For similar reasons, previous trials of severe influenza have employed a similar ordinal outcome.[43] This ordinal scale was selected as an outcome in multiple ongoing COVID-19 trials across a range of illness severity, including outpatients, and is a preferred outcome by the World Health Organization Research and Development Blueprint for COVID-19.[44] For our cohort of outpatients, we have enhanced the outcome with the addition of COVID-related symptoms to better distinguish recovery in those with milder disease. Use of this standardized outcome will increase the potential to compare the results of this trial with other trials and perform meta-analyses.

6. DATA COLLECTION

Given the infectious risk from COVID-19 and potential shortages of personal protective equipment (PPE), we will have no face-to-face contact between patients and non-clinical staff. Additionally, minimizing research activities and conducting the trial in a pragmatic manner will increase the ability to complete the trial in the face of strained clinical and research resources during the COVID-19 pandemic. We will emphasize data collection by electronic methods, supplemented by telephone or videophone follow-up and from the electronic health record. If the patient is hospitalized, we will attempt to obtain permission for accessing these records. We will not collect biological specimens as part of this trial.

Electronic Data Collection:
We will collect data directly from patients using MyCap or, if installation and use of an app is not possible, through text messaging or email with a survey link, or phone call as back up. MyCap is a secure mobile application developed by the REDCap team at Vanderbilt and integrated into the REDCap database system. Participants install the application on their personal device. All interaction with the app is secure and requires the user to enter a 6-digit pin. Once a patient has consented, they join a study for data collection by scanning a unique QR code. Then, patients receive a notification each day to answer questions about their current symptom burden, any hospitalizations, and any oxygen requirements. This provides the capacity for collecting patient reported outcomes in a secure and robust manner. If patients are unable to use this application or unwilling to install it, they can be asked the same messages using a text messaging system will be utilized. Our outcomes are designed such that a text messaging option is not overly burdensome and can even be completed in hospitalized patients. The process for using text messaging is HIPAA compliant. Finally, we can use standard telephone calls as a back-up method to administer the questions. This minimizes loss to follow up and maximizes the efficiency of the study. Research teams have had great success using REDCap-based mobile data collection for multiple studies including collection of data about quality of life and exercise after heart surgery (NCT03270124), pain reporting in sickle cell disease (NCT03629678), and pain using a visual analog scale (VAS) during an interventional drug study (NCT03865940).

Telephone/Videophone Data Collection:
While primary data collection will be by electronic methods as above, specific circumstances will trigger telephone or videophone follow-up, based on the following:

- Participant does not complete daily electronic data collection for two consecutive days.
- Participant does not complete Study Day 29 assessment
- Identification of newly prescribed medication if deemed necessary by site investigator.
- New electrocardiogram

- Lack of administration of study medication, if unable to confirm plan to resume study medication
- Hospitalization
- Reported adverse events, if deemed necessary by the site investigator
- Clarification or concern by study team
- By request of participant

6.1 Baseline Variable Collection

- Informed Consent Document and documentation of consent process
- Presence or absence of each inclusion and exclusion criterion
- Date and time of enrollment
- Day of symptom onset
- Specimen type, date, and result of SARS-CoV-2 testing conducted clinically.
- Demographics (age, sex, race, ethnicity, height, weight)
- Comorbidities: chronic cardiac disease, chronic pulmonary disease, chronic kidney disease, chronic liver disease, chronic neurological disease, malignant neoplasm, chronic hematologic disease, AIDS/HIV, obesity, diabetes with complications, diabetes without complications, rheumatologic disorder, malnutrition, smoking, other
- Chronic use of medication: ACE inhibitors, angiotensin receptor blockers
- Receipt of azithromycin in the past week
- Presenting signs and symptoms, along with self-reported severity

6.2 Daily Assessments between Randomization and Study Day 16, and then at Day 29

- Study drug administration and reason for missed doses.
- Modified COVID Ordinal Outcomes Scale
- Daily signs and symptoms, along with self-reported severity via electronic log and/or telephone interview.
- Day of symptom resolution
- Receipt of open label antivirals: chloroquine, hydroxychloroquine, remdesivir, lopinavir/ritonavir, other
- Receipt of open label immunomodulators between enrollment and hospital discharge: corticosteroids, tocilizumab, sarilumab, interferon β, other
- Receipt of azithromycin
- Study days in hospital (if applicable)
- Study days receiving supplemental oxygen (if applicable)
- Study days receiving non-invasive or invasive mechanical ventilation (if applicable)
- Study days in ICU to Study Day 29
- Study day of death (if applicable)
- Safety Outcomes: seizure, atrial or ventricular arrhythmia, cardiomyopathy, cardiac arrest, aspartate aminotransferase or alanine aminotransferase levels that are greater than twice the local upper limit of normal (or two times higher than known baseline), acute pancreatitis (defined by a clinically obtained lipase level above the local upper limit of normal and abdominal pain or vomiting), stage II or greater acute kidney injury according to KDIGO criteria[45], receipt of new renal replacement

therapy, symptomatic hypoglycemia, neutropenia, lymphopenia, anemia, thrombocytopenia, or severe dermatologic reaction (e.g., Steven's Johnson Syndrome). Since we will not systematically obtain laboratory tests to identify asymptomatic changes in laboratory values, we will ask about outpatient laboratory testing or obtain the requisite results of laboratory tests from medical records if tests were obtained.

- Prolonged (>500 msec) QTc interval on available electrocardiograms
- Self-reported adverse events to Study Day 15

7. STATISTICAL CONSIDERATIONS

Using intention-to-treat principles, we will compare the primary outcome (Modified COVID Ordinal Outcomes Scale score to Day 15) between patients in each intervention group and the control group using a proportional odds model, adjusted for the following co-variables: age, gender, and duration of acute respiratory infection symptoms prior to randomization. The proportional odds assumption will mainly be examined using graphical methods—e. g., the logit of the empirical cumulative distribution function of the ordinal scale should be parallel among categories of covariates. If proportionality is clearly violated, we will consider partial proportional odds or non-proportional odds models.

With type I error of 0.05, enrolling 540 patients in the primary population will provide 90% power to detect an odds ratio of 1.75. Accounting for a ~10% loss to follow-up rate, we will plan to enroll **600 patients**. This sample size and power assumes a frequentist analysis, which is generally more conservative than what is needed for a Bayesian approach. We will plan an initial DSMB evaluation of safety and feasibility after n=100 patients have been enrolled; efficacy will not be considered at these analyses. The trial will then have frequent interim analyses with details on specifics on timing of meetings, as well as efficacy and futility stopping rules to be detailed in the Statistical Analysis Plan, to be finalized prior to the first interim analyses.

Additional analyses will include comparisons of secondary outcomes between treatment groups. We will compare the Modified COVID Ordinal Outcome Scale daily over the 14-day intervention period using a recently developed longitudinal proportional odds model that can incorporate the information on intermediate states between baseline and Study Day 15. For time-to-event outcomes we will utilize survival models (e.g., Cox proportional hazards models), continuous outcomes will utilize linear regression, and dichotomized outcomes will be evaluated using logistic regression. We will also evaluate primary and main secondary outcomes in key subgroups of interest. *A priori* subgroups for analysis will include age, sex, race/ethnicity, facility residence, BMI, baseline renal function, hypertension, diabetes, cardiovascular disease, and duration of respiratory symptoms.

The proposed trial will use a Bayesian approach to implement adaptive elements and to estimate the primary outcome using the proportional odds model to calculate the posterior probability that the OR > 1, suggesting any benefit. Additionally, we will calculate posterior probabilities to account for the potential that a treatment is ineffective (e.g., OR < 1) or causes harm (i.e., safety concerns) and would be of consideration to terminate enrollment due to futility. A Bayesian approach provides the ability for frequent updating of the posterior probabilities to rapidly identify if a treatment is showing benefit to reducing hospitalization and severity of disease, providing the possibility of more quickly identifying beneficial treatments. The proposed design may evolve as more is learned about COVID-19 and the

treatment responses, with an adaptive Bayesian design that we expect to be more efficient and flexible being in active development.

We expect that given the circumstances of this trial during a rapidly evolving pandemic, the DSMB will need maximum latitude in modifying this trial if necessary, based on other studies of lopinavir/ritonavir that may be conducted simultaneously and the rate of growth or decline of the epidemic. Alterations may include adding patients, adding arms to the trial, declining to stop for futility, or stopping earlier to make the results public if that is necessary. We may also need to collapse categories within the Modified COVID Ordinal Scale based on granularity of data or frequency of an individual category. We are exploring other possible statistical plans that will be included in a final statistical analysis plan and will be presented to the DSMB before the first interim report.

8. DATA QUALITY MONITORING AND STORAGE

8.1 Data Quality Monitoring

We will review data quality remotely using front-end range and logic checks at the time of data entry and back-end monitoring of data using application programming interface tools connecting the online database to statistical software to generate data reports.

8.2 Data Storage

Site personnel will enter study data into a secure online database. Site personnel will maintain the data in the secure online database until the time of study publication. At the time of publication, DCC will generate a de-identified version of the database.

9. RISK ASSESSMENT

9.1 Potential Risk to Participants

Although lopinavir/ritonavir are FDA approved medications with a long history of patient use and well-established safety profiles, potential risks exist to participating in this study of lopinavir/ritonavir versus placebo for the treatment of COVID-19.

9.1.2 Potential risks of receiving lopinavir/ritonavir

The safety and tolerability of lopinavir/ritonavir has been well characterized through clinical trials and post-marketing experience since first authorization for use in 2001 for the approved HIV indication with over 7 million patient years of exposure. Based on clinical trials and post-marketing experience, the most frequently reported adverse drug reactions among patients receiving lopinavir/ritonavir were gastrointestinal disorders (including diarrhea, nausea, vomiting, and upper and lower abdominal pain), fatigue/asthenia, respiratory tract infection (upper and lower), lipid elevations (hypercholesterolemia and hypertriglyceridemia), musculoskeletal pain (including arthralgia and back pain), and headache (including migraine). Key safety concerns include metabolic abnormalities such as dyslipidemia and insulin resistance, pancreatitis, and hepatotoxicity. In addition, in the HIV population, immune reconstitution inflammatory syndrome (IRIS) manifesting as autoimmune disorders (such as Grave's disease) has been reported.

Important potential risks include PR prolongation at therapeutic dosing, and QT prolongation with supratherapeutic doses. Lopinavir/ritonavir interacts with several drugs since it is an inhibitor of the P450 isoform CYP3A and is likely to increase the plasma concentration of drugs that are metabolized by CYP3A4. Therefore, LPV/r should not be co-administered with drugs primarily metabolized by CYP3A and for which elevated plasma concentrations are associated with serious and/or life-threatening events. A list of such products is included in the lopinavir/ritonavir label. Rare reports of second- or third-degree atrioventricular block in patients with underlying structural heart disease and pre-existing conduction system abnormalities, or in patients receiving drugs known to prolong the PR interval such as verapamil, have been reported in patients receiving lopinavir/ritonavir. Lopinavir/ritonavir should be used with caution in such patients. In addition, QT prolongation with supratherapeutic doses and when lopinavir/ritonavir is co-administered with drugs known to prolong the QT interval has been reported. Because lopinavir/ritonavir is principally metabolized by the liver, caution should be exercised when administering this drug to patients with impaired hepatic function. Extra monitoring is recommended when diarrhea occurs. The relatively high frequency of diarrhea during treatment with lopinavir/ritonavir may compromise the absorption and efficacy (due to decreased compliance) of lopinavir/ritonavir or other concurrent drugs. Serious persistent vomiting and/or diarrhea with lopinavir/ritonavir use might also compromise renal function. The safety of lopinavir/ritonavir for treatment of patients with COVID-19 disease is unknown. COVID-19 may be associated with cardiac effects. lopinavir/ritonavir may prolong QT at supratherapeutic doses and prolong PR at therapeutic doses, resulting in arrhythmias.

9.1.3 Potential risks of receiving placebo with COVID-19

One potential risk to participating in this study is receiving placebo rather than lopinavir/ritonavir. This risk is only relevant if lopinavir/ritonavir are ultimately found to be an effective therapy for COVID-19 and is not relevant if lopinavir/ritonavir are both ultimately found to be ineffective therapy for COVID-19. This trial protocol minimizes this risk through rigorous design to minimize the number of patients that we must enroll to determine whether these therapies are an effective therapy for COVID-19 and allocating approximately one-third of eligible patients to placebo (instead of one half in a traditional two-arm trial). We will also exclude patients who decline to participate because they feel their optimal care requires lopinavir/ritonavir, exclude patients whose treating clinicians declines to allow enrollment because they feel the patient's optimal care requires treatment with lopinavir/ritonavir, and specify procedures for stopping the study drug, unblinding, and allowing open-label administration of lopinavir/ritonavir for patients who experience clinical deterioration during the study period.

9.2 Minimization of Risk

Federal regulations at 45 CFR 46.111(a)(1) require that risks to participants are minimized by using procedures which are consistent with sound research design. This trial protocol incorporates numerous design elements to minimize risk to patients that meet this human subject protection requirement. Lopinavir/ritonavir is approved by the U.S. Food and Drug Administration and have been used in clinical practice for decades in a number of patient populations with an established safety profile. The dose and route of administration of both medications in this trial are comparable to the dose and route of administration approved for the treatment of other acute infections, such as HIV-1. The duration of treatment in this trial of 14 days is significantly shorter than for treatment of HIV-1 infection, for which the drug is frequently administered for multiple years. To further mitigate risk, we will exclude patients

with specific risk factors for adverse events from study medication, we will exclude patients with higher risk for adverse events and patients receiving medications that may interact with lopinavir/ritonavir.

We will collect data on adverse events that are significant enough to warrant health care evaluation. It was determined that transient, and clinically insignificant changes in the electrocardiogram or laboratory testing would not be required. In addition, the additional risk to the public and to research personnel by exposing them to COVID-19 patients by requiring additional research testing is greater than the benefits of this monitoring. The trial protocol includes active monitoring of clinically significant adverse events, clinical outcomes, and interim analyses by an independent data and safety monitoring board empowered to stop or modify the trial at any time, including the need to request additional safety monitoring if determined to be warranted.

9.3 Potential Benefit

Study participants may or may not receive any direct benefits from their participation in this study. Administration of lopinavir/ritonavir may improve clinical outcomes among outpatient adults with COVID-19.

9.4 Risk in Relation to Anticipated Benefit

Federal regulations at 45 CFR 46.111 (a)(2) require that "the risks to subjects are reasonable in relation to anticipated benefits, if any, to subjects, and the importance of the knowledge that may reasonably be expected to result." Based on the preceding assessment of risks and potential benefits, the risks to subjects are reasonable in relation to anticipated benefits. Lopinavir/Ritonavir has been used in clinical practice for decades and previously evaluated for the treatment of patients acutely ill from infection with substantial data to support its safety and potential efficacy.

10. HUMAN SUBJECTS PROTECTIONS

Each study participant must sign and date an informed consent form or complete the consent in accordance with ethics committee guidelines for obtaining consent. Approval of the central institutional review board will be required before any participant is entered into the study.

10.1 Selection of Subjects

Federal regulations at 45 CFR 46(a)(3) require the equitable selection of subjects. We will screen non-hospitalized patients with positive SARS-CoV-2 testing to determine if a patient meets inclusion and exclusion criteria. Data that have been collected as part of the routine clinical care of the patient will be reviewed to determine eligibility. Study exclusion criteria neither unjustly exclude classes of individuals from participation in the research nor unjustly include classes of individuals for participation in the research. Hence, the recruitment of participants conforms to the principle of distributive justice.

10.2 Informed Consent

Federal regulations 45 CFR 46.111(a)(5) require us to seek informed consent from each patient. Study personnel obtaining informed consent are responsible for ensuring that the patient understands the risks and benefits of participating in the study, answering any questions the patient may have throughout the study and sharing any new information in a timely manner that may be relevant to the patient's

willingness to permit the patient's continued participation in the trial. The study personnel obtaining informed consent will make every effort to minimize coercion. We will inform all patients of the objectives of the study and the potential risks. We will use the informed consent document to explain the risks and benefits of study participation to the patient in simple terms before the patient is entered into the study, and to confirm that the patient is satisfied with his or her understanding of the risks and benefits of participating in the study and desires to participate in the study. The investigator is responsible for ensuring that informed consent is given by each patient. This includes obtaining the appropriate signatures and dates on the informed consent document prior to the performance of any protocol procedures including administration of study agent.

For additional details, see Section 3.6.

10.3 Withdrawal of Consent

Participating patients may withdraw or be withdrawn (by the treating physician or investigator) from the trial at any time without prejudice. Site personnel will include data up to the point of withdrawal in the trial analysis, unless consent to use data is also withdrawn. Site personnel will record consent prior to receipt of study drug, which will constitute a screen-failure. Withdrawal of consent after randomization and administration of one or more doses of study drug will lead to discontinuation of study interventions but site staff will request access to medical records for data related to the trial.

10.4 Confidentiality

Federal regulations at 45 CFR 46 111 (a) (7) requires that when appropriate, there are adequate provisions to protect the privacy of participants and to maintain the confidentiality of data. At no time during the course of this study, its analysis, or its publication will we reveal patient identities in any manner. We will collect the minimum necessary data containing patient or provider identities. All patients will be assigned a unique study ID number for tracking. We will enter all data collected for this study into a secure online database. We will maintain all data in the secure online database until the time of study publication. At the time of publication, we will generate a de-identified version of the database. Further, we will use tools within the secure online database so that only the coordinating center and investigators from the enrolling site will have access to data from participants enrolled at that site.

11. ADVERSE EVENTS

Assuring patient safety is an essential component of this protocol. Lopinavir/ritonavir is approved by the Food and Drug Administration and there is an have been used in clinical practice for decades with a well-established safety profile. Use of lopinavir/ritonavir for the treatment of acute respiratory infection due to COVID-19, however, raises unique safety considerations. This protocol addresses these considerations through:

1. Exclusion criteria designed to prevent enrollment of patients likely to experience adverse events with receipt of lopinavir/ritonavir
2. On-study monitoring of co-interventions (e.g., newly prescribed medications) and patient characteristics (e.g., healthcare utilization, clinically obtained electrocardiogram) to intervene before adverse events occur.
3. Systematic collection of safety outcomes relevant to use of lopinavir/ritonavir in this setting.

4. Structured reporting of adverse events

11.1 Adverse Event Definitions

Adverse Event: Any untoward medical occurrence associated with the use of a drug or a study procedure, whether or not considered drug related.

Serious Adverse Event: A serious adverse event is any adverse event that results in one of the outcomes listed in section 11.3 below.

Adverse Reaction: An adverse reaction means any adverse event caused by a study intervention. An adverse reaction is a subset of all suspected adverse events where there is a reason to conclude that the study intervention caused the event.

Suspected Adverse Reaction: Any adverse event for which there is a reasonable possibility that the study procedures caused the adverse event. Reasonable possibility means there is evidence to suggest a causal relationship between the study procedures and the adverse event. A suspected adverse reaction implies a lesser degree of certainty about causality than adverse reaction.

Suspected Unexpected Serious Adverse Reaction (SUSAR): An adverse reaction that is both unexpected (not consistent with risks outlined in the study protocol or investigator brochure), serious, and meets the definition of a suspected adverse reaction.

11.2 Safety Monitoring

Assuring patient safety is an essential component of this protocol. Each participating investigator has primary responsibility for the safety of the individual participants under his or her care. The Investigators will determine daily if any adverse events occur during the period from enrollment through **study day 16** (or 48 hours after completion of the study drug administration) and will determine if such adverse events are reportable. Thereafter, adverse events are not required to be reported to the IRB unless the investigator feels the adverse event was related to study drug or study procedures.

The following adverse events will be considered reportable and thus collected in the adverse event case report forms:

- Serious adverse events
- Non-serious adverse events that are considered by the investigator to be related to study procedures or of uncertain relationship (Appendix C)
- Events leading to permanent discontinuation of study drug.

Study-specific clinical outcomes (Primary, Secondary and Safety Outcomes and Assessments During the Study), including serious outcomes such as organ failures and death, are systematically recorded in the case report forms and are exempt from adverse event reporting unless the investigator deems the event to be related to the administration of study drug or the conduct of study procedures (or of uncertain relationship) as outlined in Appendix C.

After randomization, adverse events must be evaluated by the investigator. If the adverse event is judged to be reportable, as outlined above, then the investigator will report to the VCC their assessment of the

potential relatedness of each adverse event to the study drug or protocol procedure via electronic data entry. Investigators will assess if there is a reasonable possibility that the study procedure caused the event, based on the criteria outlined in Appendix C. Investigators will also consider if the event is unexpected. Unexpected adverse events are events not listed in the study protocol and the investigator brochure for Lopinavir/ritonavir. Investigators will also determine if adverse events are unanticipated given the patient's clinical course, previous medical conditions, and concomitant medications.

If a patient's treatment is discontinued as a result of an adverse event, study site personnel must also report the circumstances and data leading to discontinuation of treatment in the adverse event case report forms.

11.3 Serious Adverse Events

Serious adverse event collection begins after randomization and study procedures have been initiated. If a patient experiences a serious adverse event after consent, but prior to randomization or starting study procedures, the event will NOT be collected. Study site personnel must alert the VCC of any **serious and study procedure related** adverse event within 24 hours of awareness of the event regardless of suspected causality. The site Investigator must sign off on the initial report within three (3) days of study team's awareness of the SAE. Alerts issued via telephone are to be immediately followed with official notification on the adverse event case report form. See Appendix C for reporting timelines for serious, unexpected, study related events (SAEs) and serious, unexpected suspected adverse reactions (SUSARs)

As per the FDA and NIH definitions, a serious adverse event is any adverse event that results in one of the following outcomes:

- Death
- A life-threatening experience (that is, immediate risk of dying)
- Prolonged inpatient hospitalization or re-hospitalization

  As per http://www.fda.gov/Safety/MedWatch/HowToReport/ucm053087.htm: Report if admission to the hospital or prolongation of hospitalization was a result of the adverse event. Emergency room visits that do not result in admission to the hospital should be evaluated for one of the other serious outcomes (e.g., life-threatening; required intervention to prevent permanent impairment or damage; other serious medically important event).
- Persistent or significant disability/incapacity

  As per http://www.fda.gov/Safety/MedWatch/HowToReport/ucm053087.htm: Report if the adverse event resulted in a substantial disruption of a person's ability to conduct normal life functions, i.e., the adverse event resulted in a significant, persistent or permanent change, impairment, damage or disruption in the patient's body function/structure, physical activities and/or quality of life.

Reportable serious adverse events that may not result in death, be life-threatening, or require hospitalization may be considered serious adverse events when, based upon appropriate medical judgment, they may jeopardize the patient and may require medical or surgical intervention to prevent one of the outcomes listed in this definition.

Serious adverse events will be collected through **Study Day 16 (or** 48 hours after completion of the study drug administration), regardless of the investigator's opinion of causation.

12. Data and Safety Monitoring Board (DSMB)

The principal role of the DSMB is to assure the safety of participants in the trial. They will regularly monitor data from this trial, review and assess the performance of its operations with respect to:

- Review of adverse events
- Review of every death occurring on study
- Interim results of the study for evidence of efficacy or adverse events
- Possible early termination of the trial because of new external information, early attainment of study objectives, safety concerns, or inadequate performance
- Possible modifications in the clinical trial protocol
- Performance of individual centers

The DSMB will consist of members with expertise in acute infections, acute lung injury, emergency medicine, biostatistics, ethics, and clinical trials. Appointment of all members is contingent upon the absence of any conflicts of interest. All the members of the DSMB are voting members. The unblinded statistician will be responsible for the preparation of all DSMB and adverse event reports and may review unblinded data. The DSMB will develop a charter and review the protocol and sample consent form during its first meeting. Subsequent DSMB meetings will be scheduled in accordance with the DSMB Charter with the assistance of the VCC. When appropriate, conference calls may be held in place of face-to-face meetings. Recommendations to end, modify, or continue the trial will be prepared by the DSMB executive secretary. Recommendations for major changes, such as stopping the trial, will be communicated immediately. Other recommendations will be distributed in writing to the VCC and sIRB, which will distribute with instructions for reporting to local IRBs when appropriate.

We will comply with the principles and approaches espoused by the NIH NHLBI guidance found here:

https://www.nhlbi.nih.gov/grants-and-training/policies-and-guidelines/nhlbi-policy-data-and-safety-monitoring-extramural-clinical-studies

13. APPENDICES

Appendix A. Schedule of Events

Schedule of Events

**Description:**	**Date of Randomization**	**Days 1-14**	**Days 15-16**	**Day 29**
Confirmation of SAR-CoV-2 positive test result	X			
Home medications	X			
Verification of Inclusion/Exclusion Criteria	X			
Remote consent	X			
Randomization	X			
IP shipped to subject	X			
Self-Administered Dosing		X		
Self-Reported Symptom Survey		X	X	X
Assessment of Adverse Events		X	X	X
Modified COVID Ordinal Outcome Scale		X	X	X
Ventilator Status		X	X	X
ICU Status		X	X	X
Oxygen Status		X	X	X
Hospital Length of Stay		X	X	X
Vital Status				X
End of Study				X

Appendix B. Potential medication interactions with lopinavir/ritonavir

Medications considered to present a potential interaction with lopinavir/ritonavir, which if ordered during the 14-day study period, will prompt study personnel to discuss with treating clinicians the risk-benefit assessment of this medication and potential need for additional monitoring: flecainide, mefloquine, methotrexate, mexilitine, rifampicin, rifapentine, amiodarone, cimetidine dofetilide, phenobarbital, phenytoin, or sotalol, propafenone, astemizole, terfenadine, alfuzosin, apalutamide, ranolazine, dronedarone, rifampin, lurasidone, cisapride, pimozide, ergot-containing medicines (including dihydroergotamine mesylate, ergotamine tartrate, methylergonovine), lovastatin, simvastatin, lomitapide, sildenafil, triazolam, midazolam, rivaroxaban, fluticasone and anticoagulants or St. John's Wort.

Appendix C: Adverse Event Reporting and Unanticipated Events

As noted in section 11, investigators will report all "serious adverse events," defined as adverse events that are serious and have a reasonable possibility that the event was due to a study drug or procedure (or of uncertain relatedness), to the VCC within 24 hours. The VCC will then notify the single Institutional Review Board (sIRB).

The National/Lead Investigator at the VCC will work collaboratively with the reporting investigator to determine if a serious adverse event has a reasonable possibility of having been caused by the study drug or study procedure, as outlined in 21 CFR 312.32**(a)**(1), and below. The CC unblinded statistician will also determine if the event is unexpected for lopinavir/ritonavir. An adverse is considered "unexpected" if it is not listed in the investigator brochure or the study protocol (21 CFR 312.32(a)). If a determination is made that a serious adverse event has a reasonable possibility of having been caused by a study procedure or the study drug, it will be classified as a suspected adverse reaction. If the suspected adverse reaction is unexpected, it will be classified as a serious unexpected suspected adverse reaction (SUSAR).

To enhance safety monitoring, the VCC will report every death by coded group (and potentially unblinded group upon request) to the DSMB. The VCC will also report all unexpected deaths, serious and treatment related adverse events, and SUSARs to the DSMB, and sIRB within 7 days after receipt of the report from a clinical site. A written report will be sent to the DSMB and the sIRB within 15 calendar days. The DSMB will also review all reported adverse events and clinical outcomes during scheduled interim analyses. The VCC will distribute the written summary of the DSMB's periodic review of reported adverse events to the sIRB. The VCC will provide to AbbVie Pharmacovigilance any safety findings for lopinavir/ritonavir (without disclosing protected health information) during the conduct of the trial per contractual language.

C.1. Unanticipated Problems (UP)

Investigators must also report Unanticipated Problems, regardless of severity, associated with study procedures within 24 hours. An unanticipated problem is defined as follows: any incident, experience, or outcome that meets all of the following criteria:

- Unexpected, in terms of nature, severity, or frequency, given the research procedures that are described in the protocol-related documents, such as the IRB-approved research protocol and informed consent document; and the characteristics of the subject population being studied.
- Related or possibly related to participation in the research, in this guidance document, possibly related means there is a reasonable possibility that the incident, experience, or outcome may have been caused by the procedures involved in the research.
- Suggests that the research places subjects or others at a greater risk of harm (including physical, psychological, economic, or social harm) than was previously known or recognized.

C.2. Determining Relationship of Adverse Events to Study Drug or Study Procedures

Investigators will be asked to grade the strength of the relationship of an adverse event to study drug or study procedures as follows:

- Definitely Related: The event follows: a) A reasonable, temporal sequence from a study procedure; and b) Cannot be explained by the known characteristics of the patient's clinical state

or other therapies; and c) Evaluation of the patient's clinical state indicates to the investigator that the experience is definitely related to study procedures.

- Probably or Possibly Related: The event should be assessed following the same criteria for "Definitely Associated". If in the investigator's opinion at least one or more of the criteria are not present, then "probably" or "possibly" associated should be selected.
- Probably Not Related: The event occurred while the patient was on the study but can reasonably be explained by the known characteristics of the patient's clinical state or other therapies.
- Definitely Not Related: The event is definitely produced by the patient's clinical state or by other modes of therapy administered to the patient.
- Uncertain Relationship: The event does not meet any of the criteria previously outlined.

C.3. Clinical Outcomes that may be Exempt from Adverse Event Reporting

Study-specific outcomes of acute respiratory infection, COVID-19, and critical illness will be systematically collected for all patients in both study group and are exempt from adverse event reporting unless the investigator considers the event to be <u>Definitely or Possibly Related</u> (or of an Uncertain Relationship) to the study drug or study procedures. Examples of study-specific clinical outcomes include:

- Death not related to the study procedures.
- Neurological events
    - Seizure
- Cardiovascular events
    - Receipt of vasopressors
    - Atrial or ventricular arrhythmia
    - Cardiac arrest
- Respiratory events
    - Hypoxemia requiring supplemental oxygen
    - Acute respiratory distress syndrome
    - Receipt of mechanical ventilation
    - Receipt of extra-corporeal membrane oxygenation
- Gastrointestinal events
    - Elevation of aspartate aminotransferase or alanine aminotransferase
    - Acute pancreatitis
- Renal events
    - Acute kidney injury
    - Receipt of renal replacement therapy
- Endocrine events
    - Symptomatic hypoglycemia
- Hematologic or coagulation events
    - Neutropenia, lymphopenia, anemia, or thrombocytopenia
- Dermatologic events
    - Severe dermatologic reaction (e.g., Steven's Johnson Syndrome)

Note: A study-specific clinical outcome may also qualify as a reportable adverse event. For example, a ventricular arrhythmia that the investigator considers Definitely or Possibly Related to the study drug would be both recorded as a study-specific clinical outcome and reported as a Serious and Definitely or Possibly Related Adverse Event.

C.4. Decision tree for determining if an adverse event is reportable

Figure 1. Decision tree for determining if an adverse event is reportable

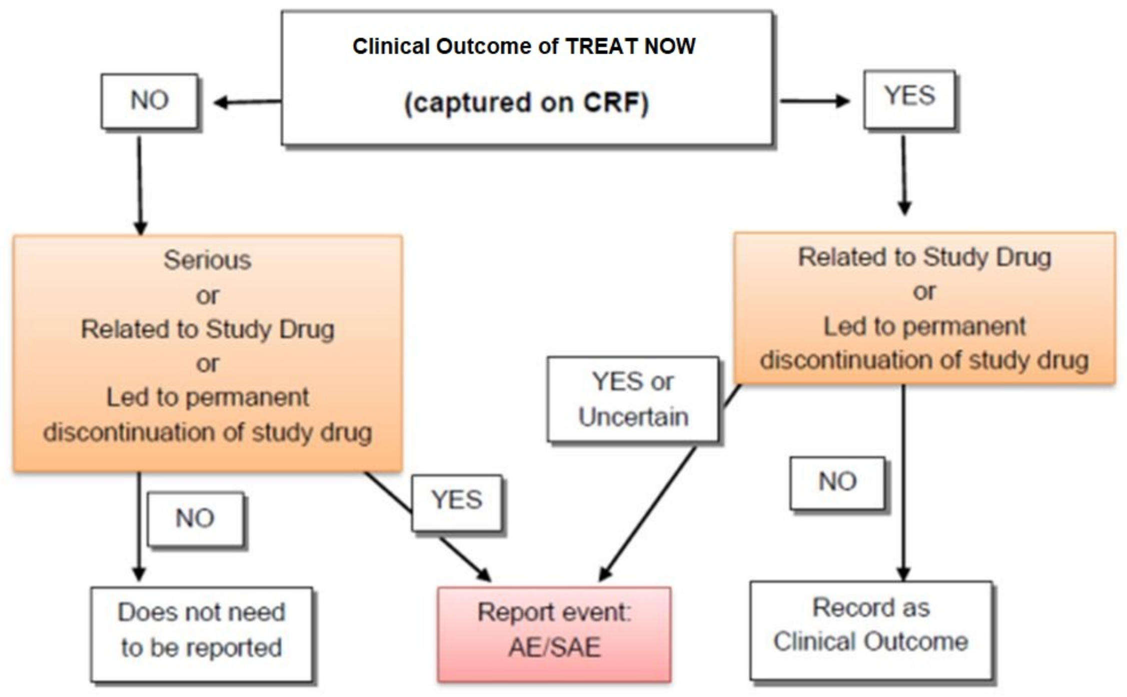

Figure adapted from Ranieri VM, Thompson BT, Barie PS et al. Drotrecogin alfa (activated) in adults with septic shock. N Engl J Med. 2012 May 31;366 (22):2055-64. PMID: 22616830.

14. REFERENCES

1. Del Rio C, Malani PN. COVID-19-New Insights on a Rapidly Changing Epidemic. JAMA 2020;

2. Fauci AS, Lane HC, Redfield RR. Covid-19 - Navigating the Uncharted. N Engl J Med 2020;

3. Cao B, Wang Y, Wen D, et al. A Trial of Lopinavir–Ritonavir in Adults Hospitalized with Severe Covid-19. N Engl J Med [Internet] 2020 [cited 2020 Apr 24];Available from: http://www.nejm.org/doi/10.1056/NEJMoa2001282

4. Wang M, Cao R, Zhang L, et al. Remdesivir and chloroquine effectively inhibit the recently emerged novel coronavirus (2019-nCoV) in vitro. Cell Res 2020;30(3):269–71.

5. World Health Organization (WHO). WHO R&D Blueprint: informal consultation on prioritization of candidate therapeutic agents for use in novel coronavirus 2019 infection, Geneva, Switzerland, 24 January 2020. [Internet]. [cited 2020 Mar 19];Available from: https://apps.who.int/iris/handle/10665/330680

6. Li Q, Guan X, Wu P, et al. Early Transmission Dynamics in Wuhan, China, of Novel Coronavirus-Infected Pneumonia. N Engl J Med 2020;

7. Zhu N, Zhang D, Wang W, et al. A Novel Coronavirus from Patients with Pneumonia in China, 2019. N Engl J Med 2020;382(8):727–33.

8. Perlman S. Another Decade, Another Coronavirus. N Engl J Med 2020;382(8):760–2.

9. Su S, Wong G, Shi W, et al. Epidemiology, Genetic Recombination, and Pathogenesis of Coronaviruses. Trends Microbiol 2016;24(6):490–502.

10. Cui J, Li F, Shi Z-L. Origin and evolution of pathogenic coronaviruses. Nat Rev Microbiol 2019;17(3):181–92.

11. Zhou P, Yang X-L, Wang X-G, et al. A pneumonia outbreak associated with a new coronavirus of probable bat origin. Nature 2020;579(7798):270–3.

12. Cheng PK, Wong DA, Tong LK, et al. Viral shedding patterns of coronavirus in patients with probable severe acute respiratory syndrome. The Lancet 2004;363(9422):1699–700.

13. Hui DS, Azhar EI, Kim Y-J, Memish ZA, Oh M, Zumla A. Middle East respiratory syndrome coronavirus: risk factors and determinants of primary, household, and nosocomial transmission. Lancet Infect Dis 2018;18(8):e217–27.

14. Wang Y, Liu Y, Liu L, Xang X, Luo N, Ling L. Clinical outcome of 55 asymptomatic cases at the time of hospital admission infected with SARS-Coronavirus-2 in Shenzhen, China. J Infect Dis 2020;

15. Wu C, Chen X, Cai Y, et al. Risk Factors Associated With Acute Respiratory Distress Syndrome and Death in Patients With Coronavirus Disease 2019 Pneumonia in Wuhan, China. JAMA Intern Med 2020;

16. Arentz M, Yim E, Klaff L, et al. Characteristics and Outcomes of 21 Critically Ill Patients With COVID-19 in Washington State. JAMA [Internet] 2020 [cited 2020 Mar 19];(Publised Online). Available from: https://jamanetwork-com.proxy.library.vanderbilt.edu/journals/jama/fullarticle/2763485

17. Wilson N, Kvalsig A, Telfar-Barnard L, Baker M. Case-fatality estimates for COVID-19 calculated by using a lag time for fatality. Emerg Infect Dis [Internet] 2020 [cited 2020 Mar 18];26(6). Available from: https://wwwnc.cdc.gov/eid/article/26/6/20-0320_article

18. Al-Bari MAA. Targeting endosomal acidification by chloroquine analogs as a promising strategy for the treatment of emerging viral diseases. Pharmacol Res Perspect 2017;5(1):e00293.

19. Chu CM, Cheng VCC, Hung IFN, et al. Role of lopinavir/ritonavir in the treatment of SARS: initial virological and clinical findings. Thorax 2004;59(3):252–6.

20. Chen F, Chan KH, Jiang Y, et al. In vitro susceptibility of 10 clinical isolates of SARS coronavirus to selected antiviral compounds. J Clin Virol 2004;31(1):69–75.

21. Wu C-Y, Jan J-T, Ma S-H, et al. Small molecules targeting severe acute respiratory syndrome human coronavirus. Proc Natl Acad Sci U S A 2004;101(27):10012–7.

22. de Wilde AH, Jochmans D, Posthuma CC, et al. Screening of an FDA-approved compound library identifies four small-molecule inhibitors of Middle East respiratory syndrome coronavirus replication in cell culture. Antimicrob Agents Chemother 2014;58(8):4875–84.

23. Chan JF-W, Yao Y, Yeung M-L, et al. Treatment With Lopinavir/Ritonavir or Interferon-β1b Improves Outcome of MERS-CoV Infection in a Nonhuman Primate Model of Common Marmoset. J Infect Dis 2015;212(12):1904–13.

24. Wang J. Fast Identification of Possible Drug Treatment of Coronavirus Disease -19 (COVID-19) Through Computational Drug Repurposing Study. J Chem Inf Model 2020;

25. Martinez MA. Compounds with Therapeutic Potential against Novel Respiratory 2019 Coronavirus. Antimicrob Agents Chemother 2020;64(5).

26. Kim UJ, Won E-J, Kee S-J, Jung S-I, Jang H-C. Combination therapy with lopinavir/ritonavir, ribavirin and interferon-α for Middle East respiratory syndrome. Antivir Ther 2016;21(5):455–9.

27. Spanakis N, Tsiodras S, Haagmans BL, et al. Virological and serological analysis of a recent Middle East respiratory syndrome coronavirus infection case on a triple combination antiviral regimen. Int J Antimicrob Agents 2014;44(6):528–32.

28. Min C-K, Cheon S, Ha N-Y, et al. Comparative and kinetic analysis of viral shedding and immunological responses in MERS patients representing a broad spectrum of disease severity. Sci Rep 2016;6:25359.

29. Chan JFW, Chan K-H, Kao RYT, et al. Broad-spectrum antivirals for the emerging Middle East respiratory syndrome coronavirus. J Infect 2013;67(6):606–16.

30. Lauer SA, Grantz KH, Bi Q, et al. The Incubation Period of Coronavirus Disease 2019 (COVID-19) From Publicly Reported Confirmed Cases: Estimation and Application. Ann Intern Med [Internet]

2020 [cited 2020 Apr 24];Available from: https://annals.org/aim/fullarticle/2762808/incubation-period-coronavirus-disease-2019-covid-19-from-publicly-reported

31. Wang D, Hu B, Hu C, et al. Clinical Characteristics of 138 Hospitalized Patients With 2019 Novel Coronavirus–Infected Pneumonia in Wuhan, China. JAMA 2020;323(11):1061.

32. Guan W, Ni Z, Hu Y, et al. Clinical Characteristics of Coronavirus Disease 2019 in China. N Engl J Med [Internet] 2020 [cited 2020 Apr 24];Available from: http://www.nejm.org/doi/10.1056/NEJMoa2002032

33. Wu C, Chen X, Cai Y, et al. Risk Factors Associated With Acute Respiratory Distress Syndrome and Death in Patients With Coronavirus Disease 2019 Pneumonia in Wuhan, China. JAMA Intern Med [Internet] 2020 [cited 2020 Apr 24];Available from: https://jamanetwork.com/journals/jamainternalmedicine/fullarticle/2763184

34. Arentz M, Yim E, Klaff L, et al. Characteristics and Outcomes of 21 Critically Ill Patients With COVID-19 in Washington State. JAMA [Internet] 2020 [cited 2020 Apr 24];Available from: https://jamanetwork.com/journals/jama/fullarticle/2763485

35. Gao J, Tian Z, Yang X. Breakthrough: Chloroquine phosphate has shown apparent efficacy in treatment of COVID-19 associated pneumonia in clinical studies. Biosci Trends 2020;14(1):72–3.

36. Woosley R, Heise C, Gallo T, Tate J, Woosley D, Romero K. www.CredibleMeds.org, QTdrugs ListCredibleMeds [Internet]. [cited 2020 Apr 24];Available from: https://www.crediblemeds.org/

37. Sapp JL, Alqarawi W, MacIntyre CJ, et al. Guidance On Minimizing Risk of Drug-Induced Ventricular Arrhythmia During Treatment of COVID-19: A Statement from the Canadian Heart Rhythm Society. Can J Cardiol [Internet] 2020 [cited 2020 Apr 24];Available from: https://linkinghub.elsevier.com/retrieve/pii/S0828282X20303251

38. Guan W, Ni Z, Hu Y, et al. Clinical Characteristics of Coronavirus Disease 2019 in China. N Engl J Med 2020;

39. Wang Y, Fan G, Salam A, et al. Comparative effectiveness of combined favipiravir and oseltamivir therapy versus oseltamivir monotherapy in critically ill patients with influenza virus infection. J Infect Dis 2019;

40. WHO | Coronavirus disease (COVID-2019) R&D [Internet]. WHO. [cited 2020 Mar 18];Available from: http://www.who.int/blueprint/priority-diseases/key-action/novel-coronavirus/en/

41. Kidney Disease Improving Global Outcomes. KDIGO clinical practice guideline for acute kidney injury. Kidney Int 2012;2: Suppl:1–138.

42. Hydroxychloroquine [Internet]. In: LiverTox: Clinical and Research Information on Drug-Induced Liver Injury. Bethesda (MD): National Institute of Diabetes and Digestive and Kidney Diseases; 2012 [cited 2020 Mar 21]. Available from: http://www.ncbi.nlm.nih.gov/books/NBK548738/
