# Supplementary material for: Trial of Early Antiviral Therapies during Non-hospitalized Outpatient Window (TREAT NOW) for COVID-19: a summary of the protocol and analysis plan for a decentralized randomized controlled trial
Source: Trials. 2022 Apr 8;23:273. doi: 10.1186/s13063-022-06213-z (PMC8990452; doi:10.1186/s13063-022-06213-z)
Supplement: Supplementary file 2 — Additional file 2. Statistical Analysis Plan. [file 13063_2022_6213_MOESM2_ESM.pdf]

Statistical Analysis Plan

We would like to acknowledge the Cambridge University Hospitals Clinical Trials Unit for the development of the template (version CCTU/TPLV2), which was modified by the Michigan Institute for Clinical & Health Research (MICHR).

TRIAL FULL TITLE	Trial of Early Antiviral Therapies during Non-hospitalized Outpatient Window (TREAT NOW) for COVID-19
SAP VERSION	1.1
SAP VERSION DATE	12/23/2021
TRIAL STATISTICIAN	Alexander Kaizer, PhD
Protocol Version (SAP associated with)	1.6
TRIAL PRINCIPAL INVESTIGATORS	Adit A. Ginde, MD, MPH<br>Nathan I. Shapiro, MD, MPH
SAP AUTHOR	Alexander Kaizer

1 SAP Signatures

I give my approval for the attached SAP for TREAT NOW dated 12/23/2021:

Statistician (Author)

Name: Alexander M. Kaizer, PhD

Signature: Alexander M. Kaizer

Date: 12/24/2021

Principal Investigators

Name: Adit A. Ginde, MD, MPH

Signature: [illegible]

Date: 12/24/2021

Name: Nathan I. Shapiro, MD, MPH

Signature: Nathan Shapiro

Date: 12/24/2021

2 Table of Contents

1 SAP Signatures ... 1
2 Table of Contents ... 2
3 Abbreviations and Definitions ... 4
4 Introduction ... 6
4.1 Preface ... 6
4.2 Scope of this statistical analysis plan ... 6
5 Study Objectives and Endpoints ... 6
5.1 Study Objectives ... 6
5.2 Treatment arms ... 6
5.3 Endpoints/Outcomes ... 7
6 Study Methods ... 9
6.1 Overview of study Design ... 9
6.2 Inclusion-Exclusion Criteria and General Study Population ... 9
6.3 Randomization and Blinding ... 9
6.4 Timing of study procedures and outcomes assessments ... 10
7 Sample Size ... 10
8 General Analysis Considerations ... 10
8.1 Timing of Analyses ... 11
8.2 Analysis Populations ... 11
8.2.1 Intention to Treat Population ... 11
8.2.2 Per Protocol Population ... 11
8.2.3 Safety Population ... 11
8.3 Covariates adjustment ... 11
8.4 Missing Data ... 12
8.5 Interim Analyses and Data Monitoring ... 12
8.5.1 Purpose of Interim Analyses ... 12
8.5.2 Planned Schedule of Interim Analyses ... 12
8.5.3 Adaptations ... 13
8.5.4 Stopping Rules ... 13
8.5.5 Considerations for Multiplicity of Testing ... 13
9 Summary of Study Data ... 14
9.1 Descriptive analysis ... 14
9.2 Subject disposition ... 15
9.3 Derived variables ... 15

9.4 Treatment compliance, protocol deviations and protocol violations 15
10 Efficacy Analyses 15
10.1 Primary Efficacy Analysis 15
10.1.1 Secondary Analyses of Primary Efficacy Endpoint 16
10.2 Secondary Efficacy Analyses 16
10.2.1 Analyses of Secondary Endpoints 16
10.3 Subgroup analyses 16
11 Safety Analyses 17
11.1 Adverse Events, Serious Adverse Events, and other Events 17
12 Reporting Conventions 17
13 References 18

3 Abbreviations and Definitions

ACE-I	Angiotensin-converting-enzyme inhibitor
ARB	Angiotensin II receptor blocker
ADR	Adverse drug reaction
ACE	Angiotensin-converting enzyme
AE	Adverse event
ALT	Alanine aminotransferase
AR	Adaptive randomization
ARDS	Acute respiratory distress syndrome
AST	Aspartate aminotransferase
AUC	Area under the curve
BAL	Bronchoalveolar lavage
BID	"Bis in die" (twice daily)
CC	Coordinating Center
CFR	Code of Federal Regulations
COVID-19	Coronavirus Disease 2019
CYP3A	Cytochrome P4503A
DCC	Data Coordinating Center
DSMB	Data and safety monitoring board
ECMO	Extracorporeal membrane oxygenation
eCRF	Electronic case report form
EKG	Electrocardiogram
FDA	Food & Drug Administration
GFR	Glomerular filtration rate
HIPAA	Health Insurance Portability and Accountability Act
HIV	Human immunodeficiency virus
HR	Hazard ratio
IC_{50}	Half maximal inhibitory concentration
ICU	Intensive care unit
ID	Identification
IL1	Interleukin-1
IL6	Interleukin-6
IP	Investigational product
IRB	Institutional Review Board
IRIS	Immune reconstitution inflammatory syndrome
IV	Intravenous
IVY Network	Influenza Vaccine Effectiveness in the Critically Ill Network
KDIGO	Kidney Disease Improving Global Outcomes
LFT	Liver function test

LPV/r	Lopinavir/ritonavir
MERS-CoV	Middle East respiratory syndrome coronavirus 2
MIC	Minimum inhibitory concentration
NHLBI	National Heart, Lung, and Blood Institute
NIH	National Institutes of Health
NSAIDs	Nonsteroidal anti-inflammatory drug
pH	Potential for hydrogen
PI	Principal investigator (a clinician responsible for one site)
PK/PD	Pharmacokinetics/pharmacodynamics
PPE	Personal protective equipment
PPoS	Predictive probability of success
QD	“quaque die” (once daily)
QID	“quater in die” (four times daily)
QR code	Quick response code
QTc	QT interval corrected for heart rate
RCT	Randomized control trial
REDCap	Research Electronic Data Capture
RT-PCR	Reverse transcription polymerase chain reaction
SAE	Serious adverse events
SARS-CoV-2	Severe acute respiratory syndrome coronavirus 2
S/F	SpO_2/FiO_2 ratio
sIRB	Single IRB
SOFA	Sequential Organ Failure Assessment
SUSAR	Suspected unexpected serious adverse reaction
US	United States
VAS	Visual Analog Scale

4 Introduction

4.1 Preface

Effective therapies for COVID-19 are urgently needed. Lopinavir/ritonavir (Kaletra), an antimicrobial agent used to treat HIV-1, has demonstrated in vitro inhibition of SARS-CoV-2, the virus that causes COVID-19. Initial clinical data have suggested possible relevance as a therapeutic agent when administered early in the disease course of patients with COVID-19. The TREAT NOW study was designed to test the hypothesis that lopinavir/ritonavir reduces disease progression and improves clinical status measured using a seven point at day 15 following receipt of study drug when compared with placebo. The TREAT NOW study is a multicenter, blinded, non-matching placebo-controlled, randomized clinical trial to evaluate the superiority of active therapy (lopinavir/ritonavir) versus placebo control. Briefly, patients testing positive for SARS-CoV-2 infection in an outpatient setting are contacted by research coordinators and invited to participate. Adults with a positive SARS-CoV-2 test are either self-referred or identified via review of testing records at participating sites. Patients who are still symptomatic and who are within six days of symptom onset are invited to participate. Eligible participants who provide their consent to participate using an electronic system undergo comprehensive baseline data collection via phone survey, the participant is randomized, and study drug or placebo is shipped overnight to the participant's home. The participant then completes a daily web-based medication adherence, symptom burden, and hospitalization survey for sixteen days. Missed surveys result in telephone follow up to complete missing data and to re-engage the participant in completing the study. At day 15, the primary outcome is assessed. Patients are followed up once more at 29 days

4.2 Scope of this statistical analysis plan

The TREAT NOW clinical protocol was developed to adapt to allow for addition of new agents and changing standards of care. In the absence of any planned adaptations, this document is restricted to the statistical design and analysis of a comparison between two groups of participants: patients randomized to receive lopinavir/ritonavir and patients randomized to receive a placebo. If new agents are introduced for testing, or other adaptations are sought, this document will be revised accordingly.

Section 5 introduces and defines the study objectives and endpoints. Study methods including the overall design, inclusion/exclusion criteria, randomization, and blinding are in Section 6. Sample size justifications are in Section 7. Section 8 discusses the analysis populations and planned interim analyses. Subject disposition, derived variable definitions, and protocol deviations are defined in Section 9. Sections 10 and 11 detail the planned efficacy and safety analyses, respectively. The final sections, after Section 12, note reporting conventions, any updates to the SAP and/or protocol after SAP approval, and references.

5 Study Objectives and Endpoints

5.1 Study Objectives

To determine whether early treatment with lopinavir/ritonavir improves clinical status at 15 days when compared to placebo in outpatient adults with COVID-19. Secondary objectives include describing the safety profile of lopinavir/ritonavir, and to determine whether the clinical progression is improved through to Day 29.

5.2 Treatment arms

The active treatment arm of lopinavir/ritonavir (LPV/r) consists of standard dosing with 400mg/100mg taken orally twice daily for 28 doses. Medication dose packs contain all 28 doses labelled by study day. The LPV/r dosing is based upon extensive PK/PD study evaluations in healthy and HIV+ subjects.

The control arm consists of unmatched placebo taken orally twice daily for a total of 28 doses. Medication dose packs will contain all 28 doses labelled by study day. Given the unmatched placebo pill to LPV/r, exclusion will be exercised to ensure two individuals from the same household are not enrolled in the study and that telephone assessments during the study avoid discussion of the appearance of the study medication.

5.3 Endpoints/Outcomes

In this section endpoints/outcomes are described with how they should be operationalized for analysis. Additional information on the study population(s) are defined in Section 8.2 with respect to those included for analysis. Per the protocol, timing of study procedures is based on the time of randomization, which is defined as Baseline. Study Day 1 is defined as the day of receipt of the first dose of the study drug.

Primary outcome:
The primary outcome is the daily status based on the proposed Modified COVID Ordinal Outcomes Scale from Study Day 1 through Study Day 15:

1. Death
2. Hospitalized on mechanical ventilation or ECMO
3. Hospitalized on supplemental oxygen
4. Hospitalized not on supplemental oxygen
5. Not hospitalized with symptoms and limitation in activity
6. Not hospitalized with symptoms but with no limitation in activity
7. Not hospitalized without symptoms nor limitation in activity

Each daily status is derived primarily from the daily study survey through day 16 after randomization and the chart review on day 29 after randomization. This is a longitudinal measure of the ordinal outcome from receipt of study medication (Study Day 1) to completion of study treatment (Study Day 15).

Secondary outcomes:
The secondary outcomes are described below with details on operationalization:

- Modified COVID Ordinal Outcome Scale on Study Day 8
    - The ordinal outcome of the previously defined scale on Study Day 8 after one week of treatment.
- Modified COVID Ordinal Outcome Scale on Study Day 15
- Modified COVID Ordinal Outcome Scale on Study Day 29
    - The ordinal outcome of the previously defined scale on Study Day 29 after one month of treatment receipt.
- Proportion of patients hospitalized through Study Day 29
    - A dichotomous indicator if the participant was hospitalized at any point through Study Day 29 as reported on daily surveys or through the Day 29 Chart Review.
- Time to hospitalization through Study Day 29
    - The number of days from receipt of study medication (Study Day 1) to first

hospitalization for any reason through Study Day 29.

- Time to symptom resolution through Study Day 29
    - The number of days from receipt of study medication (Study Day 1) to resolution of all symptoms reported without recurrence. For example, if a participant reports no symptoms on the daily survey on Study Day 5, but then symptoms are reported again on Study Days 6 through 8 with resolution of all symptoms on Study Day 9, the number of days for resolution would be 9 and not 5 to reflect the recurrence of symptoms.
- All-cause, all-location mortality up to Study Day 29
    - A binary variable indicating death for any reason from receipt of study drug (Study Day 1) through Study Day 29.
- Oxygen-free days through Study Day 29
    - The number of days derived from the Modified COVID Ordinal Outcome Scale where there is no reported supplemental oxygen, mechanical ventilation, or ECMO from receipt of study drug (Study Day 1) to Study Day 29 during hospitalization. Death before Study Day 29 will be penalized by using a value of -1 free days.
- Fever-free days through Study Day 29
    - The number of days where fever is reported as “None” versus “Mild”, “Moderate”, or “Severe” based upon daily surveys between receipt of study drug (Study Day 1) and Study Day 29. Death before Study Day 29 will be penalized by using a value of -1 free days.
- Ventilator-free days through Study Day 29
    - The number of days derived from the Modified COVID Ordinal Outcome Scale where there is no reported mechanical ventilation or ECMO from receipt of study drug (Study Day 1) to Study Day 29. Death before Study Day 29 will be penalized by using a value of -1 free days.
- Vasopressor-free days through Study Day 29
    - The number of days from receipt of study drug (Study Day 1) to Study Day 29 based upon the Study Day 29 Chart Review where no vasopressor use is denoted. Death before Study Day 29 will be penalized by using a value of -1 free days.
- ICU-free days through Study Day 29
    - The number of days from receipt of study drug (Study Day 1) to Study Day 29 based upon the Study Day 29 Chart Review where there is no portion of the day spent in the ICU. Death before Study Day 29 will be penalized by using a value of -1 free days.
- Hospital-free days through Study Day 29
    - The number of days from receipt of study drug (Study Day 1) to Study Day 29 based upon daily surveys and the Study Day 29 Chart Review where there is no portion of the day spent admitted to a hospital setting. Death before Study Day 29 will be penalized by using a value of -1 free days.

Safety outcomes:
Safety outcomes to be included in the safety analysis for this trial include all potentially associated adverse events (PAAEs) as well as other events of interest including, but not necessarily limited to:

- Seizure
- Atrial or ventricular arrhythmia
- Cardiac arrest
- Receipt of renal replacement therapy

- Severe dermatologic reaction
- *Elevation in aspartate aminotransferase or alanine aminotransferase to twice the local upper limit of normal AND at least doubling over known baseline
- *Acute pancreatitis
- *Acute kidney injury by KDIGO criteria
- *Symptomatic hypoglycemia
- *Anemia or thrombocytopenia

* For participants whose symptoms are significant enough to trigger a clinical work-up and thus have clinically available testing

6 Study Methods

6.1 Overview of study Design

The study is a multicenter, blinded, non-matching placebo-controlled, randomized clinical trial to evaluate the superiority of active therapy (lopinavir/ritonavir) versus placebo control. Patients, treating clinicians, and study personnel are blinded to study group assignment. Randomization uses permuted blocks and is stratified by site and age (≥65 years or <65 years) in a 1:1 ratio through a central electronic system. The primary outcome, measured from Study Day 1 (receipt of study drug) to Study Day 15, is a seven-level ordinal scale describing the patient's clinical status defined in Section 5. The planned analysis is a longitudinal Bayesian proportional odds model with a skeptical prior, and is powered to detect an odds ratio of greater than 1.75.

6.2 Inclusion-Exclusion Criteria and General Study Population

Patients aged ≥18 years who have laboratory-confirmed SARS-CoV-2 infection by RT-PCR or other molecular test within the past 6 days, whose symptoms began ≤6 days previously, and who currently remain with at least one symptom are eligible to participate. Qualifying symptoms are cough, fever, shortness of breath, chest pain, abdominal pain, nausea/vomiting, diarrhea, body aches, weakness/fatigue.

Detailed exclusion criteria are listed in the protocol. They include known contraindications for lopinavir/ritonavir, clinical conditions increasing the risk of adverse events associated with lopinavir/ritonavir, known pregnancy or breast feeding, known to be a prisoner, inability to comply with study procedures, and inability to provide informed consent or complete daily symptom surveys in English or Spanish during the two-week observational period.

6.3 Randomization and Blinding

Eligible patients will be randomized in a 1:1 ratio to the intervention arm (lopinavir/ritonavir) or the control arm (placebo). Randomization is stratified by site and age (≥65 years versus <65 years) and uses permuted small block randomization. Patients, treating clinicians, trial personnel, and outcome assessors are blinded to group assignment. Only the investigational pharmacy (who is distributing study medication), the Data Coordinating Center (who is managing the randomization systems including generating the randomization sequence), and one member of the statistical team (who is preparing closed DSMB interim reports) are unblinded.

To maintain the blind, study medication is dispensed with packaging and labelling that does not indicate whether the drug is a placebo or active treatment. Unblinding will occur only if required for subject safety or treatment at the request of the treating clinician and will be done by the

Investigational Pharmacy.

6.4 Timing of study procedures and outcomes assessments

The day of randomization is defined as Baseline and the receipt of the study drug is defined as "Study Day 1". It is possible for it to take up to two days for study medications to arrive at a participant's home. For the purposes of analysis, Study Day 1 will be counted as the day on which study medications are received by the participant. To ensure symptoms are assessed daily while on study, and to ensure the primary outcome can be measured through Study Day 15, symptoms are collected daily from the day following randomization to sixteen days following randomization. The symptoms assessed on Study Day 1 (day of receipt of medication) to Study Day 15 are included in the measurements for this trial. At Study Day 15, the primary outcome is assessed. At Study Day 29, additional outcomes are assessed.

7 Sample Size

This study is designed to be analyzed using a Bayesian approach, accepting the possibility of adaptations or sample size adjustments as the trial progresses. Although the design as detailed is Bayesian in nature, a frequentist sample size and target power are provided for consistency with FDA guidance and recommendations. To provide a sample size estimate, we calculated a sample size of 540 patients equally distributed among study arms is needed to have 90% power to detect an odds ratio of 1.75 or greater assuming a traditional proportional odds model and a type I error of 0.05, as proposed by Whitehead (1993). Accounting for an approximate 10% loss to follow-up rate, we plan to enroll 600 patients

An example of the difference between study arms resulting in such an odds ratio is shown in the table. This is based on recommendations from the World Health Organization for COVID-19 Master Protocols that were in place at the time this study was designed

Category	Placebo Arm Proportion	Treatment Arm Proportion
1-Death	1%	0.6%
2-Hospitalized on mechanical ventilation or ECMO	3%	1.8%
3-Hospitalized on supplemental oxygen	4%	2.4%
4-Hospitalized not on supplemental oxygen	3%	1.9%
5-Not hospitalized with symptoms and limitation in activity	10%	6.6%
6-Not hospitalized with symptoms but with no limitation in activity	24%	18.7%
7-Not hospitalized without symptoms nor limitation in activity	55%	68.1%

8 General Analysis Considerations

Here, we describe overarching guidelines that will be followed in the statistical analysis of the TREAT NOW study. The timing and scope of each analysis, the analysis datasets, the approach to handling

missing data, covariate adjustment, subgroup analysis and stopping guidelines are provided. In subsequent sections, the approach to describing the data and modeling the outcomes of interest are described.

8.1 Timing of Analyses

The final analysis will be performed after all subjects enrolled have completed their Day 29 data collection or dropped out prior to their Day 29 data collection, and data are declared query free. Interim analyses are detailed below for safety, efficacy, and futility and will be performed for the primary outcome based on a database pull for interim analyses. At conclusion of the trial and after resolution of all queries, the database will be locked.

8.2 Analysis Populations

The analysis populations are defined below. A participant's inclusion or exclusion status with regard to each analysis population will be set prior to breaking the blind for final analyses based on the criteria as outlined.

8.2.1 Intention to Treat Population

- All subjects who have receipt of study drug (Study Day 1) based upon their randomized allocation.
- Given the study design whereby patients are shipped study drug from a central pharmacy, there might be times when the drug does not get delivered to the patient. In these rare instances the patient will be excluded. All other subjects will be included, and they will be analyzed according to which group the patient was assigned. Thus, the intent to treat (ITT) analysis set includes all subjects who were randomized and received the study drug at their provided address.

8.2.2 Per Protocol Population

- All subjects who receive study drug, report taking at least one dose, and who complete their Study Day 15 survey will be included in the per protocol population.
- Patients will be classified according to the treatment they received with confirmation of receipt from the study pharmacy.

8.2.3 Safety Population

- All subjects who received any study treatment (including control) and reported taking at least one dose but excluding subjects who drop out prior to receipt of the study drug. In the unlikely case a participant receives the incorrect study drug, patients will be grouped according to the treatments that they received in any safety analyses. If a participant receives both placebo and active treatment, they will be considered as receiving active treatment.

8.3 Covariate adjustment

The main analysis of the primary outcome will be a covariate adjusted proportional odds model. Covariates that will be adjusted for include

- Race/ethnicity defined as a single categorical variable for non-Hispanic White/Caucasian, non-Hispanic Black/African American, Hispanic, Other.
- Age as a continuous variable in years; modeling will assess for linear effect or cubic splines based on the study data.
- Gender as a categorical variable for male, female, undifferentiated, and no answer
- Duration of acute respiratory infection symptoms prior to study drug receipt in days calculated as the duration reported at enrollment for randomization plus the additional days

until receipt of study drug

- Presence of comorbidities as a dichotomous variable for presence of any or absence of all of the following: hypertension, diabetes mellitus, obesity, chronic kidney disease, chronic cardiopulmonary conditions, or immunosuppressive conditions.
- Monoclonal antibodies taken for the treatment of COVID-19 prior to randomization defined as a binary variable (yes/no)
- SARS-CoV-2 vaccinations were added to the data collection on May 14th, 2021. After that time, enrolled subjects will be categorized as fully vaccinated (at least 14 days after 2nd dose of SARS-CoV-2 mRNA vaccine or after single dose of J&J vaccine prior to randomization); partially vaccinated (receipt of one or more doses of SARS-CoV-2 vaccine but not fully vaccinated prior to randomization); and not vaccinated. Between December 14th, 2020 (first day of public vaccination in the US under EUA) and May 13th, 2021, vaccination status will be recorded as missing. Prior to December 14th, 2020, vaccination status will be recorded as not vaccinated.
- Time period when date of randomization occurred defined as a categorical variable in quarters (3 month) periods beginning with June 2020-August 2020, September 2020-November 2020, December 2020-February 2021, etc.

Center effects will be accounted for with the specification of a random intercept. The defined groupings above may be modified to collapse groups pending the available data at time of final study analysis.

8.4 Missing Data

Missing outcome data for an analysis population will be imputed when necessary using multiple imputation. Outcomes using the Bayesian longitudinal proportional odds model, which includes the primary outcome, will not use imputation since the model facilitates smoothing over missing responses (i.e., the model can still be used even if a participant has missing day(s) of response data). Data on age, gender, race/ethnicity, comorbidities, and duration of acute respiratory infection prior to randomization should not be missing as they are collected as part of enrollment, however imputation procedures will be used in cases of missing values.

8.5 Interim Analyses and Data Monitoring

8.5.1 Purpose of Interim Analyses

Interim analyses are planned for safety, efficacy, and futility. The results of the interim analyses will be reported to the DSMB, along with the stopping guidelines described in this Statistical Analysis Plan. The DSMB may request additional interim analyses. Such requests will be fulfilled by the unblinded statistician and will not be reported outside of the DSMB meeting.

8.5.2 Planned Schedule of Interim Analyses

The first interim analysis is planned after at least the first 50 participants have been enrolled and achieved follow-up through at least Study Day 15. This first analysis will present only safety data to the DSMB. The second and subsequent interim analyses will include both safety and efficacy data, and are planned to occur after at least 200 participants have been enrolled and have their primary outcome observed at Study Day 15. Futility interim analyses will begin after approximately 400 participants have been observed and will be conducted in addition to interim analyses for safety and efficacy.

This trial is planned to be analyzed using a Bayesian approach, and so a strict interim analysis schedule is not prespecified. Instead, the schedule for subsequent interim analyses will be decided by the DSMB based upon the rate of accrual, the safety and feasibility data from at least the first 200

participants, opportunities to add new agents, and other relevant considerations.

8.5.3 Adaptations

The statistical design of this study is sufficiently flexible to allow for future adaptations. However, no adaptations are currently planned. Should new agents become available for inclusion in the study, or should there be sufficient evidence to warrant changes in the planned randomization allocation probabilities or sample size, the adaptations will be encoded in a revision to this document before being implemented.

8.5.4 Stopping Rules

Given the Bayesian nature of the analysis, the *posterior probability* of the odds ratio for the primary outcome exceeding certain thresholds will be used to guide decisions to stop the trial early for efficacy or harm. These are:

Probability	Evidence For	Action Trigger
P_1 = P(OR > 1 | data)	Any benefit	$P_1 > 0.95$
P_2 = P(OR < 1 | data)	Inefficacy or potential harm	$P_2 > 0.90$

The criteria for P_1 represents scenarios where an intervention arm would stop early for efficacy, whereas P_2 represents early termination of an intervention arm for inefficacy or potential harm.

Interim monitoring for futility will be based on the *posterior predictive probability* that P(concluding efficacy at N=300 per group|interim data). For the calculation of PPoS, the efficacy threshold on study completion will be based on the posterior probability that P(OR > 1 | data) > 0.90. The posterior predictive probability of success (PPoS) will be estimated via upstrapping the remaining number of participants to be enrolled from participants who have completed through Study Day 15 regardless of level of missing data (Crainiceanu and Crainiceanu 2020). The PPoS will be based on 1,000 upstraps of the “completed” trial with stopping rules based on the following table:

Predictive Probability of Success (PPoS) Action Trigger	Action
PPoS < 0.10	Stop for futility
0.10 $\le$ PPoS < 0.25	Consider stopping for futility based on trial characteristics (e.g., accrual), secondary endpoints, and external factors
PPoS $\ge$ 0.25	Continue trial

8.5.5 Considerations for Multiplicity of Testing

Unlike a frequentist design with confidence intervals and p-values relating to the unknown parameters, a Bayesian design conditions on the data to estimate the unknown parameters. Therefore, the type of control for multiplicity used in a frequentist approach is inappropriate. However, it is not uncommon to attempt to understand the frequentist operating characteristics for Bayesian designs. We note that a 5% type I error rate with identical assumptions to detect an OR=1.75 achieves 86.4% power with a target sample size of 600. With regards to repeated evaluations of efficacy using the Bayesian approach, the posterior probability is an estimate of the unknown parameter conditional on the data collected thus far and any prior estimates of the posterior probability are irrelevant, and so multiplicity adjustments are not warranted and would be inappropriate.

9 Summary of Study Data

All continuous variables will be summarized using the following descriptive statistics: n (non-missing sample size), mean, standard deviation, median, minimum, 1st quartile, 3rd quartile, and maximum. All categorical variables will be reported as the frequency and percentages of observed levels with a clear definition of the denominator. In general, summary tables will be based on the relevant population (e.g., ITT, safety) and structured with a column for each study arm and will be annotated with the total sample size relevant to that table/treatment, including the number of missing observations.

9.1 Descriptive analysis

Using data pooled across all sites, the study sample will be characterized based on demographic and clinical variables measured at randomization, unless otherwise indicated. Specifically, the following variables will be described:

1. Age (years)
2. Sex (male, female, undifferentiated, no response)
3. Race (Black/African American, White/Caucasian, American Indian/Alaskan Native, Asian, Native Hawaiian/Other Pacific Islander, other, no answer)
4. Ethnicity (Hispanic, non-Hispanic)
5. Race/ethnicity (non-Hispanic Black/African American, non-Hispanic White/Caucasian, Hispanic, other)
6. Body mass index (kg/m^2)
7. Presence of comorbidities (yes/no):
    a. chronic cardiac disease
    b. chronic pulmonary disease
    c. chronic kidney disease
    d. chronic liver disease
    e. chronic neurological disease
    f. malignant neoplasm
    g. chronic hematologic disease
    h. AIDS/HIV
    i. obesity
    j. diabetes with complications
    k. diabetes without complications
    l. rheumatologic disorder
    m. malnutrition
    n. immunosuppressive conditions
8. Tobacco product use (current, former, never)
9. E-cigarette or vaping product use (current, former, never)
10. Chronic use of medication (yes/no):
    a. ACE inhibitors
    b. angiotensin receptor blockers
11. Receipt of azithromycin in the past week
12. Presence of symptoms (yes/no):
    a. Cough
    b. Fever
    c. Shortness of breath
    d. Chest pain
    e. Abdominal pain

   f. Nausea
   g. Diarrhea
   h. Body aches
   i. Weakness/fatigue
13. Total number of symptoms reported (count)
14. Symptom duration (days)

9.2 Subject disposition

The number of participants completing each stage of the trial will be summarized with frequencies and percentages to describe the number screened, the number randomized, the number reaching target data collection times by arm at Study Day 8, Study Day 15 (primary), and Study Day 29 for collection of the primary ordinal outcome status, and the number that dropped out by arm and the accompanying reason where known (e.g., death, toxicity, withdrew consent). A CONSORT diagram will be generated (Schulz et al. 2010).

9.3 Derived variables

This study involves several derived variables, such as body mass index and the seven-level ordinal outcome scale. During the data collection process, each component of the derived variable is collected and the first stage in data analysis will be to generate and validate the derived variable. Depending on the distribution of the seven-category modified COVID-19 ordinal outcomes scale, certain categories may need to be combined based upon few or no events.

The primary outcome through Study Day 15 will be derived primarily through the daily surveys (from Days 1 to 16 post-randomization) and supplemented by the Study Day 29 Chart Review to verify details about hospitalization or other healthcare encounters recorded. The method for derivation of other variables not described will be clearly articulated in the analysis report if needed.

9.4 Treatment compliance, protocol deviations and protocol violations

Major protocol deviations and violations will be described. Adherence to the schedule for taking study medications will be reported. Major protocol deviations that will be reported are failure of participant to adhere to the study medication schedule, errors in shipping and handling of study medication including shipping the incorrect medication, and other protocol deviations deemed by the study executive committee to affect the overall integrity of the data. Any such determination will be made before unblinding to treatment arm.

10 Efficacy Analyses

All continuous and categorical variables will be summarized as described previously. The analytic approach is Bayesian with results presented as credible intervals and posterior probabilities for specific hypotheses as defined in the following sections unless otherwise noted.

10.1 Primary Efficacy Analysis

The primary outcome of the Modified COVID Ordinal Outcomes Scale from Study Day 1 through Study Day 15 will be evaluated in the ITT dataset using a Bayesian longitudinal proportional odds model. The model will include a random intercept for each participant to account for the repeated measurements over time to calculate the posterior probability that a treatment arm compared to the placebo-controlled arm has an OR > 1, suggesting any benefit for the treatment. The proportional odds assumption will be examined using graphical methods—e. g., the logit of the empirical cumulative distribution function of the ordinal scale should be parallel among categories of

covariates. If proportionality is clearly violated, we will consider partial proportional odds or non-proportional odds models.

The model will be adjusted for the variables described in Section 8.3. To account for changes in the effect size between treatment groups from Study Day 1 to Study Day 15, an interaction between randomized group and time will be included. Time will initially be modeled using a cubic spline, but it may be modified to a simpler or more complex representation of time if needed to appropriately model the effect over time.

The prior for the intercept for the proportional odds regression model will assume a Dirichlet distribution. The treatment effect, as defined as a log odds ratio in the model, will have a normal prior defined so that $P(OR > 1) = P(OR < 1) = 0.5$ (i.e., equally as harmful as beneficial) and $P(OR > 2) = P\left(OR < \frac{1}{2}\right) = 0.025$ (i.e., large effects in either direction unlikely), with the variance computed to satisfy these criteria. For all other covariates the prior distributions will be normal with mean 0 and a larger variance to reflect the uncertainty of their potential effect.

The convergence of the Markov chain Monte Carlo simulations will be checked via diagnostics, with alternative priors, models, and chain lengths/burn-ins explored if necessary to achieve convergence and satisfy any model assumptions.

10.1.1 Secondary Analyses of Primary Efficacy Endpoint

We will replicate the primary analyses using the per protocol dataset and the safety dataset.

10.2 Secondary Efficacy Analyses

10.2.1 Analyses of Secondary Endpoints

For time-to-event outcomes we will utilize survival models (e.g., Cox proportional hazards models), and dichotomized outcomes will be evaluated using logistic regression. The continuous outcomes such as hospital free days are penalized for death and so an ordinal model will be fit in preference to using linear regression. Secondary endpoint models will be fit using normal priors with mean 0 and larger variance to reflect the uncertainty of their potential effect on all regression coefficients, with convergence evaluated similarly to the primary endpoint.

10.3 Subgroup analyses

The currently recommended approach to subgroup analysis is to prespecify subgroups where there is a clear biological rationale, and otherwise not to create subgroups unless the data indicate differential treatment effects based on potential subgrouping variables. Given how little is known about COVID-19 and possible treatment response, we will examine the potential of differential treatment effects in key subgroups of interest based upon previous definitions unless otherwise noted:

- Age
- Sex
- Race/ethnicity
- BMI
- Baseline renal function defined as known severe chronic kidney disease requiring dialysis, kidney disease not requiring dialysis, no known history of kidney disease
- Hypertension
- Diabetes defined as diabetes with complications, diabetes without complications, not diabetic

- Cardiovascular disease
- Duration of respiratory symptoms prior to randomization

Interactions will not be tested together within the same model but will be tested one by one. Potentially significant subgroups will be identified by evaluating the posterior weight assigned to a model with and without the interaction by Bayesian stacking. If the model including the interaction has a posterior weight of 80% or greater, we will proceed with fitting the model within subgroups. For continuous variables, graphical methods will be used to show how the treatment effect changes over the range of the continuous variable.

11 Safety Analyses

The safety analysis set will be used for safety analyses. Summary statistics will be calculated as described in Section 9. When calculating the incidence of adverse events, or any sub-classification thereof by treatment, time period, severity, etc., each subject will only be counted once for a given safety outcome and any repetitions will be reported separately; the denominator will be the total safety population size.

Three tables summarizing adverse events will be created:

- All-Cause Mortality: A table of all anticipated and unanticipated deaths due to any cause, with number and frequency of such events in each arm/group of the clinical study.

- Serious Adverse Events: A table of all anticipated and unanticipated serious adverse events, grouped by organ system, with number and frequency of such events in each arm/group of the clinical study.

- Other (Not Including Serious) Adverse Events: A table of anticipated and unanticipated events (not included in the serious adverse event table) that exceed a frequency threshold of 5% within any arm of the clinical study, grouped by organ system, with number and frequency of such events in each arm/group of the clinical study.

11.1 Adverse Events, Serious Adverse Events, and other Events

The summary statistics will be produced in accordance with section 9. Adverse events, serious adverse events, and other events are defined in Appendix C of the study protocol. The discontinuation of study medication due to potential adverse events or side effects will be summarized by self-reported termination of study treatment and reported adverse events or side effects.

12 Reporting Conventions

For the purposes of the statistical analysis plan report, posterior probabilities and p-values ≥0.001 will be reported to 3 decimal places; values less than 0.001 will be reported as “<0.001”. The mean, standard deviation, and any other statistics other than quantiles, will be reported to one decimal place greater than the original data. Quantiles, such as median, or minimum and maximum will use the same number of decimal places as the original data. Estimated parameters, not on the same scale as raw observations (e.g. regression coefficients) will be reported to 3 decimal places.

13 References

Crainiceanu CM and Crainiceanu A. The upstrap. *Biostatistics* 21.2 (2020): e164-e166.

Harrell F. rmsb: Bayesian Regression Modeling Strategies. R package version 0.0.1. (2020): https://CRAN.R-project.org/package=rmsb

Schulz KF, Altman DG, Moher D, CONSORT Group. CONSORT 2010 Statement: Updated Guidelines for Reporting Parallel Group Randomised Trials. *PLoS Med* 7.3 (2010): e1000251.

Whitehead J. Sample size calculations for ordered categorical data. *Statistics in medicine* 12.24 (1993): 2257-2271.
